# Ultra-high molecular weight linear coordination polymers with terpyridine ligands†
†Electronic supplementary information (ESI) available. See DOI: 10.1039/c9sc01115c


**DOI:** 10.1039/c9sc01115c

**Published:** 2019-05-15

**Authors:** Reece W. Lewis, Nino Malic, Kei Saito, Richard A. Evans, Neil R. Cameron

**Affiliations:** a Department of Materials Science and Engineering , Monash University , 22 Alliance Lane , Clayton , Victoria 3800 , Australia . Email: neil.cameron@monash.edu; b CSIRO Manufacturing Flagship , Clayton , 3168 , Australia . Email: richard.evans@csiro.au; c School of Chemistry , Monash University , Clayton , 3800 , Australia; d School of Engineering , University of Warwick , Coventry , CV4 7AL , UK

## Abstract

Ultra-high molecular weight (UHMW, *M*_n_ > 1000 kDa) polymeric drift control adjuvants (DCAs) for agricultural spraying are prone to mechanical degradation and rapidly lose performance. To overcome this, we have designed linear coordination polymers (LCPs) composed of 400 kDa telechelic bis-terpyridine end-functionalised polyacrylamide units, which ‘self-heal’ upon shearing through reformation of coordination bonds. After addition of Fe(ii) to dilute aqueous solutions of the terpyridine telechelics, UHMW LCPs were obtained as demonstrated by UV-vis spectroscopy, MALS GPC and intrinsic viscosity measurements. Importantly, these UHMW LCPs were shown to function as effective DCAs, reducing the formation of fine ‘driftable’ droplets during spray testing at concentrations as low as 100 ppm. Following mechanically-induced coordination bond-scission, the UHMW LCPs were found to recover up to 90% of their performance compared to un-sheared samples, at a rate dependent on the transition metal ion used to form the complex.

## Abstract 2

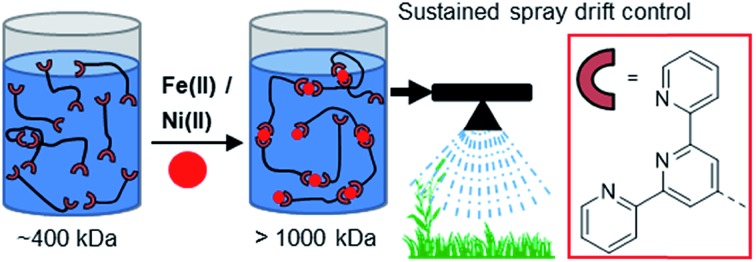
This first report of ultra-high molecular weight (>1000 kDa) linear coordination polymers demonstrates their use in agricultural spray drift control.

## Introduction

Ultra-high molecular weight (UHMW, *M*_n_ > 1000 kDa) linear polymers are able to increase solution extensional viscosity at concentrations as low as 100 ppm, with application in mist control,^1^ drag reduction^2^,^3^ and reducing agrichemical spray drift.^4^,^5^ Extensional stresses along the elongated chain can also cause mechanical degradation of the polymer (covalent bond scission), with longer chains being more susceptible due to a greater accumulation of stress.^6^ This presents a problem, as low molecular weight (degraded) polymers have reduced extensional viscosity effect and performance. For example, spray drift during agrichemical spraying can cause damage to neighbouring crops and the environment; and is more likely to occur for sprays with fine, ‘driftable droplets’ (generally < 150 μm in diameter).^7^,^8^ UHMW polyacrylamide and polyethyleneoxide drift control adjuvants (DCAs) have been shown to initially reduce formation of fine droplets, however after repeated circulations through a centrifugal pump (causing mechanical degradation) they were found to have similar performance to water alone.^9^

To overcome this loss of performance for DCAs, we investigated if linear coordination polymers (LCPs) could associate into UHMW species in dilute aqueous solution, and if so, reversibly break and reassociate after shearing. Due to the dynamic property of LCPs, their molecular weight is a function of many variables including concentration, which complicates comparisons across literature. However, in general, molecular weights of LCPs have been limited to the order of 100 kDa at concentrations of 5–20 mg mL^–1^.^10^–^13^ The molecular weight of LCPs can be calculated by multiplying the molecular weight of the bi-functional monomer with the average degree of polymerisation (DP). The DP can be crudely described by a function of the concentration (*c*) and association constant (*K*_a_) (eqn (1)),^14^,^15^ however many other factors not accounted for in this equation (such as end-group fidelity) can have an effect.^16^

1 DP ∼ (*K*_a_ × *c*)^1/2^

Under dilute conditions the formation of cyclic structures or rings is increasingly favoured, which can act as chain stoppers and reduce LCP molecular weight.^17^,^18^ Smaller ring structures are more likely to form than larger ones, with flexible linkers just large enough to form a single unstrained ring being particularly susceptible to ring formation.^19^ Short, bulky linkers which are unable to self-cyclise have thus been widely used to limit the formation of cyclic species.^20^,^21^

Limiting cyclic species formation with high molecular weight linker units (>100 kDa) has been less explored, and utilises the idea that rings are less likely to form from larger chains due to an increase in the entropic penalty of cyclisation.^22^ This strategy was successfully implemented by Kornfield *et al.* investigating the formation of shear stable polymers for reducing jet fuel (organic non-polar solvent) misting. Ultra-high molecular weight ‘megasupramolecules’ were synthesised by end-functionalising large (up to 670 kDa) poly(1,5-cyclooctadiene) linkers with hydrogen bonding units, resulting in the dilute solution (2 mg mL^–1^) formation of supramolecular polymers of *M*_w_ ≈ 2200 kDa.^1^ This work indicates that development of our proposed analogous water-soluble system using coordination bonds, which unlike hydrogen bonds are not significantly weakened in polar protic solvents such as water, may have application in agricultural spray drift control.^23^,^24^ To outperform conventional covalent polymers, the LCPs would need to undergo selective mechanically-induced dissociation and re-association in solution, which, outside of the work conducted by the group of Sijbesma on the successful reformation of smaller phosphine-based LCPs after sonication, has not been widely studied.^25^–^29^ Terpyridine ligands have been attached to low molecular weight linker units (≤25 kDa) for LCP synthesis and form coordination complexes of wide ranging stability constants and kinetic properties (Table 1),^10^,^12^,^30^–^32^ which may allow for optimisation and improved understanding of the reformation process.

**Table 1:** Key physical property data for terpyridine complexes used in this study

Constant	Unit	Fe(ii)	Cu(ii)	Zn(ii)	Ni(ii)	Co(ii)
Stability constant (tpy mono-complex; aq. 25 °C)	Log(K1)	7.1 (ref. 33)	12.3 (ref. 34)	6 (ref. 33)/8.2^*a*^ 35	10.7 (ref. 33)	9.5 (ref. 33)/8.4 (ref. 36)
Stability constant (tpy bis-complex; aq. 25 °C)	Log(K2)	13.8 (ref. 33)	6.8 (ref. 34)	6.1^*a*^ 35	11.1 (ref. 33)	9.1 (ref. 33)/9.9 (ref. 36)
Ligand exchange half-life ([M tpy_2_]^2+^ with tpy)	(min)	8400 (ref. 37) (35.4 °C), 2730 (ref. 37) (45.8 °C)	<0.1 (ref. 37) (0.1 °C)	<0.1 (ref. 37) (0.1 °C)	610 (ref. 37) (44.8 °C)	19 (ref. 37) (10.5 °C)
Formation rate constant (mono-complex)	Log(M^–1^ s^–1^)	4.75 (ref. 36) (25 °C)	7.30 (ref. 36) (6.5 °C)	6.04 (ref. 36) (25 °C)	3.15 (ref. 36) (25 °C)	4.38 (ref. 36) (25 °C)
Formation rate constant (bis-complex)	Log(M^–1^ s^–1^)	7.04 (ref. 36) (5 °C)	—	—	4.88 (ref. 36) (10 °C)	6.70 (ref. 36) (5 °C)
*M*_n,th_ at 1 mg mL^–1^ of 400 kDa telechelic^*d*^	kDa	11 000^*b*^	406^*b*^	401^*b*^	7250^*b*^	924^*b*^
		4720^*c*^	406^*c*^	401^*c*^	3900^*c*^	867^*c*^

^*a*^Measurements made in acetonitrile.

^*b*^Assuming 100% end-group fidelity.

^*c*^Assuming 95% end-group fidelity.

^*d*^Details of stability constant calculations to determine *M*_n,th_ are provided in Scheme S1 and Table S1.

Herein we report the first UHMW LCP formed in dilute aqueous solution through the use of high molecular weight (∼400 kDa) polyacrylamide linker species end-functionalised with terpyridine ligands. The telechelic polymers were synthesised by RAFT polymerisation of acrylamide (AM) with bis terpyridine-functionalised CTAs, and the resultant LCPs were characterised by GPC, viscometric, screen factor, spray testing and shearing analysis.

## Results and discussion

### RAFT agent synthesis

With the desire to form UHMW LCPs from high molecular weight (∼400 kDa) coordination ligand end-functionalised telechelics, an appropriate means of synthesising such telechelics with high end-group fidelity was needed. When functionalising polymers of high molecular weight, post-polymerisation modification becomes increasingly problematic due to non-quantitative conversion, exacerbated by the high dilution of end-groups.^38^ Therefore, use of reversible addition–fragmentation chain transfer (RAFT) polymerisation^39^,^40^ with a functional chain transfer agent (CTA) was investigated.

**Scheme 1:**
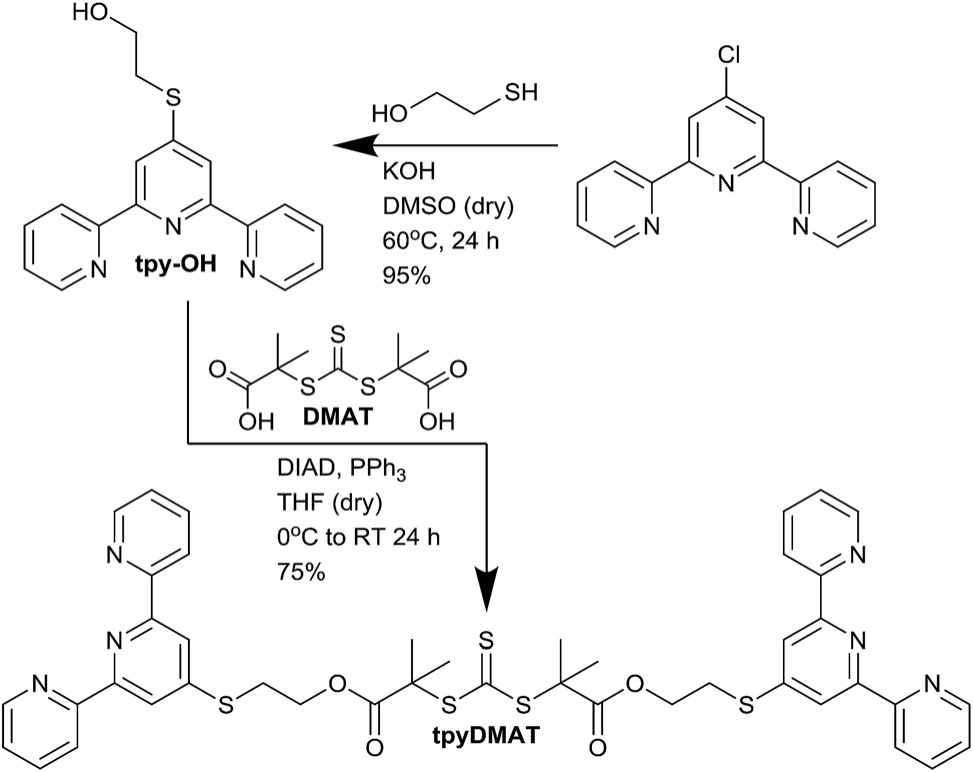
Synthesis of new bis-terpyridine functionalised RAFT agent (tpyDMAT). Full synthetic details in ESI.†

The previously reported bis-terpyridine CTA bis(4′-(*p*-methylenephenyl)-2,2′:6′,2′′-terpyridine) trithiocarbonate (tpyTTC) was then synthesised according to a modified literature procedure (Scheme S2†).^30^ Initial attempts at polymerising AM with tpyTTC proved challenging and prompted the synthesis of a new CTA to produce comparative telechelic polymer samples. On the basis of our^41^ and other research groups^42^,^43^ success with the symmetrical trithiocarbonate DMAT for AM polymerisations, a new bis-terpyridine RAFT agent (tpyDMAT) was synthesised by esterification of DMAT with a terpyridine functionalised alcohol (tpy-OH, Scheme 1). The formation of both tpy-OH and tpyDMAT was supported by ^1^H NMR, ^13^C NMR and mass spectroscopies. Both tpyTTC and tpyDMAT were then utilised for AM telechelic polymer synthesis to determine which CTA was better suited for such application.

### Telechelic polymer synthesis

**Scheme 2:**
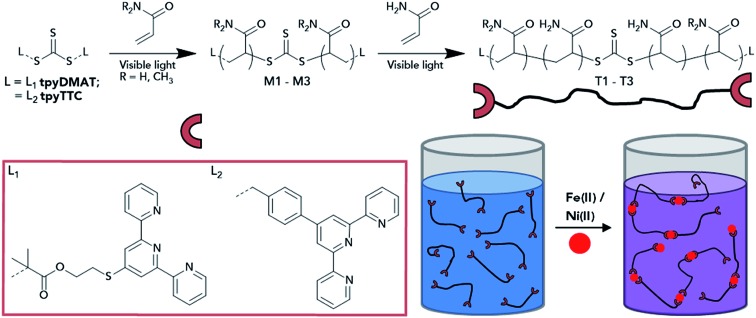
Telechelic terpyridine end-functionalised polymer synthesis and subsequent association into LCPs after Fe(ii) or Ni(ii) complexation.

Polyacrylamide is only soluble (particularly at high molecular weights) in water, where both terpyridine RAFT agents are insoluble. To overcome this, macro-CTAs were first synthesised with either AM or *N*,*N*-dimethylacrylamide (DMA) monomer in DMSO (where polyacrylamide is soluble at low molecular weights), before chain-extending with AM to approximately 400 kDa (Scheme 2). Both macro-CTA and chain-extension polymerisations were initiated by photolysis of the RAFT agent under visible light irradiation (DP-RAFT),^44^ which we have found is well-suited to high molecular weight (*M*_n_ > 1000 kDa) AM polymerisations.^41^ In addition, since this technique avoids the use of an external initiator, formation of unwanted α termini from the radical source is avoided.^45^

**Fig. 1:**
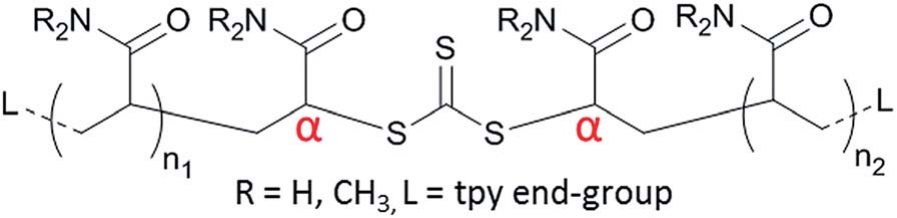
General structure of macro-CTAs synthesised in this study with the α-proton labelled.

In attempting to achieve a high end-group fidelity after AM chain-extensions to 400 kDa, it was discovered that careful optimisation of the macro-CTA synthesis conditions was required. Many different combinations of monomer, solvent, RAFT agent and irradiation intensity were trialled, and the most successful conditions resulted in the macro-CTAs M1–M3 (Table 2). Successful macro-CTA synthesis was primarily assessed by the integral of the ^1^H NMR α-proton signal (4.3–5.1 ppm), which is expected to integrate to 2 when each of the terpyridine aromatic proton signals is set to 4 (Fig. 1). Macro-CTAs with an α-proton integral significantly less than 2 were adjudged to have formed significant amounts of mono-telechelic type polymers which can act as chain stoppers, suppressing LCP growth (Fig. S3–S5†).

**Table 2:** Polymerisation conditions and characterisation data for terpyridine end-functionalised macro-CTAs

Code	CTA	[Monomer]: [CTA]	Polym time (h)	Light power	Conv. (%)	*M*_n,conv._ (kDa)	*M*_n,NMR_ (kDa)	α-Proton integral
M1^*a*^	tpyDMAT	17 (AM)	1.5	52 W	79	1.81	1.82	1.98
M2^*a*^	tpyDMAT	32 (DMA)	1	39 W	97	4.29	4.57	1.80
M3^*a*^ ^,^^*b*^	tpyTTC	74 (DMA)	3	26 W	74	6.27	7.01	1.91

^*a*^Macro-CTAs were synthesised in DMSO, under 402 nm irradiation.

^*b*^Polymerisation was cooled to 20 °C, with 0.18 M PTSA to dissolve tpyTTC. ^1^H NMR spectrum for each macro-CTA in Fig. S1–S3.

Initial chain extensions of M1 to low molecular weights (∼15 kDa) demonstrated the utility of tpyDMAT to successfully form terpyridine end-functionalised polymers by RAFT polymerisation, as determined by GPC and ^1^H NMR spectroscopic analysis (Fig. S6a and b†). They were also found to form LCPs after complexation with Fe(ii) (Fig. S6c and d†), however as expected they were of insufficient molecular weight for spray drift application. For this purpose, three ∼400 kDa terpyridine functionalised telechelic polymers (T1–T3, Table 3) were synthesised by chain-extending M1–M3 (respectively) with AM ([AM] : [CTA] = 7000). Monomodal distributions were recorded after aqueous RI GPC analysis, and despite high dispersities (*Đ* = 1.53–2.44) which may in part be due to end-group column interactions (Fig. S7†), further characterisation of these polymers indicated successful formation of the desired terpyridine end-functionalised species.

**Table 3:** Polymerisation conditions and characterisation data for high molecular weight terpyridine end-functional polymers^*a*^

Code	(Macro) CTA	Polym time (h)	Conv. (%)	*M*_n,conv._ (kDa)	*M*_n,GPC_ (kDa)	*Đ*	*M*_n,UV_ ^*b*^ (kDa)
T1	M1	15	72	362	158	1.59	542 ± 2
T2	M2	8.6	73	368	174	1.53	455 ± 7
T3	M3	6.6	85	428	121	2.44	570 ± 53

^*a*^T1–T3 were synthesised in H_2_O with either TFA or HCl to adjust the pH to 3.2–3.7, under 444 nm (12 W) irradiation, with [AM] : [CTA] = 7000. The small addition of acid was implemented as initial attempts at approximately neutral pH indicated that ester hydrolysis (on tpyDMAT polymers) was leading to poor retention of end-groups.

^*b*^The error value reported is the SD across absorbance data for that polymer. Calculation used *ε*_566_ = 9536 cm^–1^ M^–1^ for T1 Fe(ii) and T2 Fe(ii) and *ε*_568_ = 11 740 cm^–1^ M^–1^ for T3 Fe(ii), full details for calculation are provided in ESI.

For example, additions of Fe(ii) to these polymers formed the expected purple colouration due to formation of the Fe(ii) bis-terpyridine complex (*λ*_max_ = 566–568 nm). The Fe(ii) complex was chosen for initial study as it produces a strong visible absorbance clear of other signals and has high stability constants, making it likely to ultimately facilitate successful association into UHMW LCPs. By measuring the peak absorbance value and assuming an end-group fidelity of 100%, an *M*_n,UV_ value was calculated for all polymers (extinction coefficients measurements presented in Fig. S8–S10†), which was in reasonable agreement with those determined by conversion (Table 3). In addition, T3 was also analysed by a Multi Angle Light Scattering (MALS) GPC (for absolute molecular weight measurements) which recorded an *M*_n,GPC_ of 551 kDa (*Đ* = 1.28), in close agreement with *M*_n,UV_ (570 kDa).

### Linear coordination polymer formation studies

**Fig. 2:**
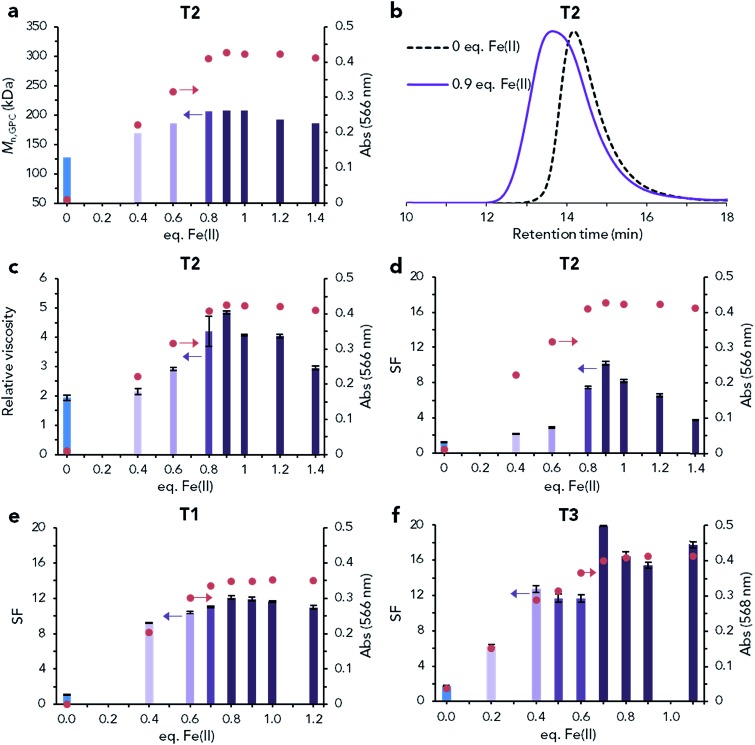
Analysis of linear coordination polymer formation after Fe(ii) addition. (a–d) Increasing equivalents of Fe(ii) were added to separate samples of T2 (at 10 mg mL^–1^) and analysed by UV-vis absorbance at 566 nm (circles, 10 mg mL^–1^) as well as: GPC (diluted to 1 mg mL^–1^) (a) *M*_n,GPC_ (bars) and (b) GPC trace of T2 un-complexed (0 eq. Fe(ii)) and fully Fe(ii) complexed (0.9 eq. Fe(ii)); (c) relative viscosity (bars, diluted to 2 mg mL^–1^, average of two independent measurements ± STD); and (d) SF (bars, diluted to 1 mg mL^–1^, average of three measurements ± STD). (e, f) Effect of addition of increasing equivalents of Fe(ii) to T1 ((e), at 10 mg mL^–1^) and T3 ((f), at 10 mg mL^–1^) on SF (bars, diluted to 1 mg mL^–1^, average of two measurements ± STD) and UV-vis absorbance at 566 nm (circles, 10 mg mL^–1^). GPC data for also collected during the titration as is presented in Fig. S12.†

Preliminary experiments on T2 were conducted to confirm that the stoichiometric equivalence of Fe(ii) determined by UV-vis spectroscopic monitored titrations, resulted in the highest molecular weight supramolecular species. Increasing equivalents of Fe(ii) were added to separate 4 mL solutions of T2 (10 mg mL^–1^), which were then analysed by UV-vis spectroscopy and diluted for relative viscosity, GPC and screen factor (SF) measurements (Fig. 2a–d). This array of molecular weight characterisation was conducted in part to determine the suitability of SF measurements (commonly performed as part of polymeric DCA testing) to UHMW LCP analysis. This technique exploits the phenomenon that polymer solutions with increased extensional viscosity (generally due to the presence of UHMW linear polymers) will resist the extensional flow created in the screen pack, causing an increased elution time.^4^,^46^

An increase in all measurements related to molecular weight (*M*_n,GPC_, relative viscosity and SF) was observed with increasing equivalents of Fe(ii) until the peak absorbance at 566 nm was recorded. This point corresponds to the experimentally determined stoichiometric equivalence, and further additions of Fe(ii) beyond this point resulted in a reduction in molecular weight. This reduction is expected, as overtitration of Fe(ii) increasingly favours the formation of mono-complexes, reducing the supramolecular DP.^16^ Of the three molecular weight measurements (which all recorded the same trend), SF measurements were identified as particularly suitable due to ease of measurement and high sensitivity (approximately 8 times increase in SF from un-complexed to fully complexed, compared to ≤2.5 times increase for relative viscosity and *M*_n,GPC_).

After establishing the utility of UV-vis spectroscopic monitored titrations, other (generally more labile) complexes of T2 were synthesised using the same titration method. Strong qualitative agreement with the trends in *M*_n,th_ calculated from stability constants for the association of dilute (1 mg mL^–1^) 400 kDa terpyridine end-functionalised telechelics was observed (Table 1). The Ni(ii) complex of T2 resulted in LCPs of similar molecular weight to the Fe(ii) species, while minimal (Co(ii)) to no (Cu(ii), Zn(ii)) increase in molecular weight was observed for the other complexes (Fig. S11†). This confirms that the stronger Fe(ii) and Ni(ii) terpyridine complexes are needed to associate high molecular weight species in dilute solution (≤10 mg mL^–1^). For simplicity, studies on the formation of UHMW LCPs continued to focus on the Fe(ii) complexes, with the Ni(ii) complex used later during shear recovery experiments.

To compare the propensity of T1–T3 to form UHMW LCP species, Fe(ii) titrations monitored by SF measurements and UV-vis spectroscopy were also performed on T1 and T3. Similar to T2, both T1 and T3 were found to have an increased molecular weight upon Fe(ii) addition, with this maximum occurring at approximately the equivalence point determined by absorbance at 566 nm (Fig. 2e and f). Of the three telechelic polymers, the largest increase in molecular weight was observed for T3, with a peak SF of 20 after Fe(ii) complexation. The deviation from an increase in SF with increasing Fe(ii) addition between 0.4 and 0.6 equivalents may be related to inhomogeneities during mixing (and LCP formation) due to the extreme extensional viscosities encountered at 10 mg mL^–1^ for these species (Fig. S13†).

**Scheme 3:**
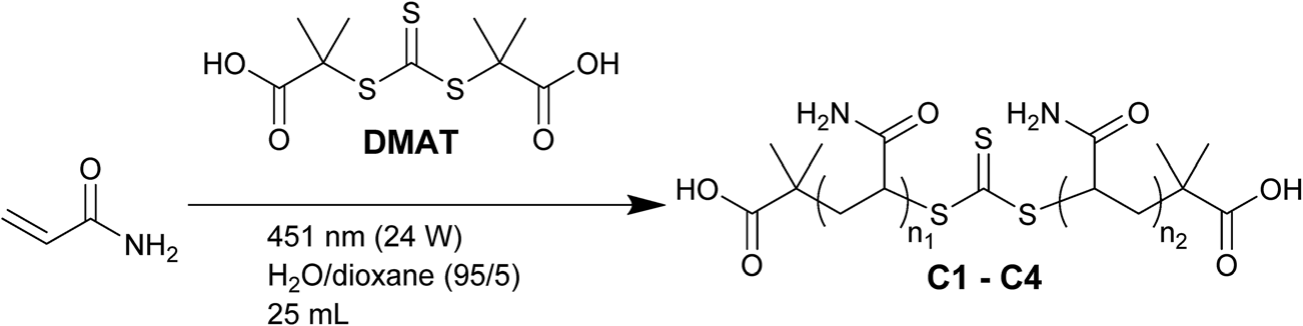
Synthesis of high molecular weight carboxylic acid end-functional polymers.

To assist molecular weight characterisation of the LCPs, control polymers were synthesised by RAFT polymerisation with DMAT (carboxylic acid end-functionalised) as the CTA (C1–C4, Scheme 3). Since these are not expected to associate into larger species in aqueous solution, they served as useful benchmarks in viscosity and other physical property analysis. DPs (at 100% conversion) between 1 and 8 times of what was implemented for the terpyridine end-functional polymers (DP = 7000) were targeted. These were then characterised by two different aqueous GPCs, confirming the formation of successively higher molecular weight species (Table 4). The lower *M*_n,GPC_ values measured on the Waters RI system compared to the Shimadzu-Wyatt MALS system may be due to the increasing presence of species beyond the Waters RI PEO calibration (∼1 × 10^6^ Da) as the target DP was increased.

**Table 4:** Characterisation data for high molecular weight carboxylic acid end-functional polymers^*a*^

Code	Polym time (h)	Conv. (%)	*M*_n,conv._ (kDa)	*M*_n,GPC_ ^*b*^ (kDa)	*Đ* ^*b*^	*M*_n,GPC_ ^*c*^ (kDa)	*Đ* ^*c*^	*M*_w,visc_ ^*d*^ (kDa)
C1	21	89	444	275	1.38	468	1.08	397
C2	24	81	806	310	1.64	633	1.08	611
C3	26	76	1520	504	1.47	815	1.11	763
C4	23	69	2750	820	1.50	1100	1.25	1480

^*a*^Each polymer was synthesised using DMAT as the CTA, H_2_O/dioxane (95/5) as the solvent under 451 nm (24 W) irradiation.

^*b*^Waters aqueous GPC (RI detector) full GPC data in Fig. S14.

^*c*^Shimadzu-Wyatt GPC (MALS detector), d*n*/d*c* evaluated to be 0.159 mL g^–1^.

^*d*^ *M*_w,visc_ evaluated from intrinsic viscosity measurements.

In addition to the series of narrow MWD RAFT polymer samples, a commercial (free radical) polyacrylamide labelled as 5000–6000 kDa (AM-5-6M) was also used as a benchmark, representative for the type of polyacrylamide currently used in polymeric DCA formulations. This was characterised by the Waters aqueous GPC, highlighting its broad MWD nature (*M*_n,GPC_ = 350 kDa, *Đ* = 2.59).

**Fig. 3:**
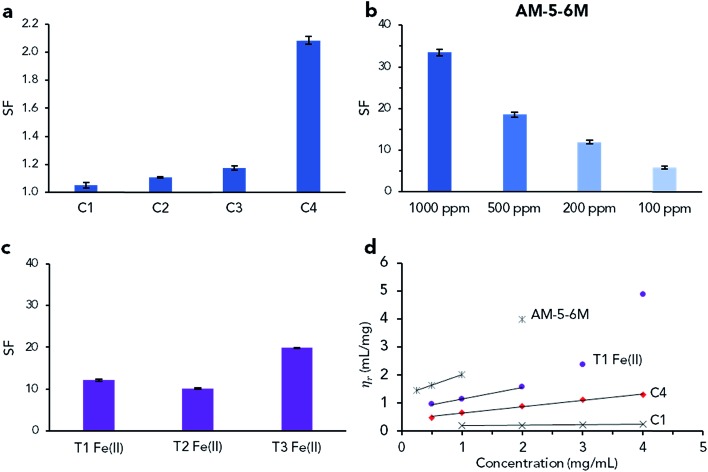
Screen factor and molecular weight characterisation of control covalent and Fe(ii) coordination polymers. (a) SF analysis of carboxylic acid end-functional control polymers (1000 ppm, average of three measurements ± STD). (b) SF analysis of AM-5-6M at 100–1000 ppm (average of three measurements ± STD). (c) UHMW LCPs formed from T1–T3 complexed with Fe(ii) at 10 mg mL^–1^ and analysed at 1000 ppm (average of three measurements ± STD). (d) Reduced specific viscosity plot of selected polymers between 0.25–4 mg mL^–1^ to determine viscosity molecular weight (C2 and C3 in Fig. S15, intrinsic viscosity data in Table S3†).

The control RAFT polymers were then analysed by SF measurements at 1 mg mL^–1^ (Fig. 3a), where little difference in SF between C1 and C3 was observed (SF = 1.0–1.2). Only the highest molecular weight sample (C4) recorded a noticeably increased value (SF = 2.1), demonstrating the limitations of SF analysis to differentiate between non-UHMW samples. Conducting SF analysis on samples of AM-5-6M, the presence of UHMW chains (≫ 1000 kDa) was seen to have a clear impact, with SF values significantly higher than C4 at 100 to 1000 ppm (SF = 5.8–33, Fig. 3b). For reference, the peak SF values recorded for the Fe(ii) LCPs (T1 Fe(ii), T2 Fe(ii) and T3 Fe(ii)) are also presented (SF = 10–20, Fig. 3c), all of which were far in excess of that recorded by the highest molecular weight control (C4, SF = 2.1). Assuming that the higher SF values recorded for the Fe(ii) LCPs are due to the presence of higher molecular weight species than in C4, a minimum supramolecular DP of 3–6 for each LCP can be estimated (depending on which *M*_n_ value is used to compare C4 with the T1–T3).

From these SF results it appears that (after careful optimisation of polymerisation conditions) both tpyDMAT and tpyTTC RAFT agents are capable of synthesising ∼400 kDa acrylamide polymers of the required end-group fidelity to associate into UHMW (*M*_n_ > 1000 kDa) species in dilute solution after Fe(ii) complexation. This conclusion was supported by further characterisation on T1 Fe(ii) and T3 Fe(ii) (complexed at 10 mg mL^–1^).

Intrinsic viscosity analysis, as has been previously used to evaluate the molecular weight of terpyridine coordination polymers,^10^,^12^ was conducted on T1 Fe(ii) and the control polymers (Fig. 3d). Intrinsic viscosity ([*η*]) is evaluated by extrapolating the reduced viscosity (*η*_r_) of a sample to zero concentration and is related to the polymer molecular weight (*M*_w,visc_) by the Mark–Houwink–Sakurada equation. The constants of this equation depend on the polymer, with the values for polyacrylamide presented in eqn (2).^47^

2 [*η*] = 1.00 × 10^–2^*M*_w,visc_^0.755^

It was found that for the control RAFT polymers (C1–C4), *M*_w,visc_ was in qualitative agreement with *M*_n,conv._ (Table 4), while for AM-5-6M there was strong agreement with the quoted molecular weight (5000–6000 kDa, *M*_w,visc_ = 5750 kDa). Importantly, *M*_w,visc_ for T1 Fe(ii) was 2180 kDa, indicating the presence of species with supramolecular DP ≥ 4, in agreement with the SF results. The formation of UHMW species for T3 Fe(ii) was supported by absolute molecular weight (MALS GPC) analysis, which recorded a shift from *M*_n,GPC_ = 551 kDa (*Đ* = 1.28) to *M*_n,GPC_ = 1056 kDa (*Đ* = 1.16) after Fe(ii) complexation.

### Spray droplet size analysis

**Fig. 4:**
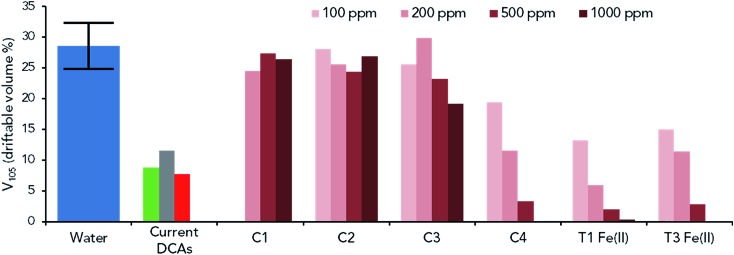
Spray driftable volume percent analysis of (from left to right): water (8 repeats), samples representative of current DCAs (green = paraffinic oil at 0.5 vol%, grey = guar at 0.5 vol%, red = AM-5-6M at 100 ppm), control polymers (C1–C4, between 100 and 1000 ppm) and LCPs T1 Fe(ii) and T3 Fe(ii) (between 100 and 1000 ppm). Additional spray data in Fig. S17–S19.†

To confirm the applicability of UHMW LCPs to spray drift application, spray droplet size measurements were conducted on T1 Fe(ii) and T3 Fe(ii). Many different parameters can be used to describe spray droplet size distributions (Fig. S16†), and for this study the *V*_105_ (vol% of droplets sprayed with diameter less than 105 μm) was used to quantitate the volume of a spray particularly susceptible to drift. Three different common types of commercial DCAs (paraffinic oil, guar and high molecular weight polymer (AM-5-6M)) were first tested at standard application concentrations and were found to reduce the *V*_105_ from 29% (from water alone, SD of 3.8%) to 8–12% (Fig. 4, current DCAs).

Solutions of the control RAFT polymers (100–1000 ppm) were then analysed under the same conditions (Fig. 4), with C1 and C2 found to have minimal effect on the droplet size distribution and *V*_105_ at all concentrations. At 500 to 1000 ppm, C3 was beginning to have a clear effect, while C4 (the highest molecular weight sample) was able to significantly reduce *V*_105_ at all concentrations (1000 ppm not tested). This data indicates that a minimum molecular weight of approximately 1000 kDa is needed to effectively reduce formation of fine droplets, in general agreement with values reported in the literature for polyethylene oxide based DCAs (minimum of 300–500 kDa).^48^,^49^

The LCPs T1 Fe(ii) and T3 Fe(ii) were then analysed (from 100 to 1000 ppm, complexes formed at 10 mg mL^–1^) and clear reductions in the *V*_105_ were observed at all concentrations, with similar performance to that of C4 (Fig. 4). At 1000 ppm, a *V*_105_ of 0.36% was recorded for T1 Fe(ii), however in practice such a high concentration would likely not be used as the simultaneous increase in the formation of large droplets would be detrimental to plant coverage. At 200 ppm (which had a much milder effect on large droplet production), T1 Fe(ii) and T3 Fe(ii) were found to either outperform or match the commercial DCAs with *V*_105_ reduced to 6–11%, respectively. This data strongly confirms that these coordination polymer systems are able to form UHMW linear polymer species, which remain present even under extreme dilutions of 100 ppm.

### Shear recovery of linear coordination polymers

Having demonstrated that UHMW LCPs are able to form from high molecular weight (∼400 kDa) terpyridine end-functionalised polymers complexed to Fe(ii), their ability to recover from shear degradation was compared to conventional UHMW covalent polymers. Preliminary shearing experiments identified that shearing AM-5-6M solutions (100 mL, 100–1000 ppm) in an Ultra-Turrax mixer at 3200 RPM for 60 minutes or 5000 RPM for 15 minutes resulted in a 77–91% reduction in SF (Fig. S20†).

The effect of these shearing treatments was then examined on both T1 Fe(ii) and T3 Fe(ii) at 1000 ppm. Solutions were prepared by diluting the un-complexed polymer to 1000 ppm, then adding in the required amount of metal ion. This dilute addition of metal ion was conducted so that the initial un-sheared SF measurements of the LCP would be conducted at equilibrium state, representative of the theoretically ‘fully recovered’ polymer. After an initial SF measurement, the LCP solutions were then divided into three samples; one which would remain un-sheared, one for shearing at 5000 RPM for 15 minutes and one for shearing at 3200 RPM for 60 minutes. Immediately after shearing the samples were analysed by SF again to characterise initial degradation, then further measurements over time were conducted to evaluate the rate of recovery.

**Fig. 5:**
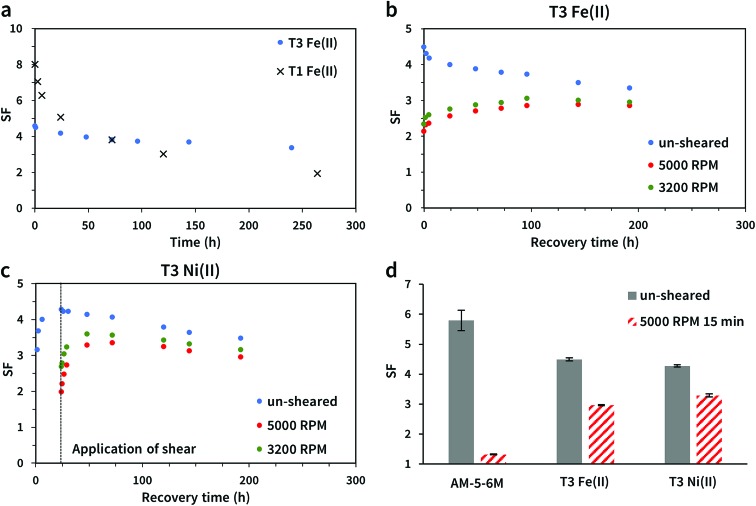
Shear recovery analysis of UHMW LCPs. (a) Comparison of reduction in SF over time for un-sheared samples of ester (T1) and ester free (T3) LCPs. (b, c) SF for solutions (1000 ppm) of sheared and un-sheared UHMW LCPs ((b): T3 Fe(ii), (c): T3 Ni(ii)) measured over 196 hours. Shear treatment was either 5000 RPM for 15 minutes or 3200 RPM for 60 minutes (100 mL solution), samples were stored in the dark between analysis. (d) Comparison of SF results before and after shearing at 5000 RPM (15 minutes) between AM-5-6M (100 ppm), T3 Fe(ii) (1000 ppm, 5 days recovery for sheared sample), and T3 Ni(ii) (1000 ppm, 1 day after recovery for sheared sample).

Experiments on T1 Fe(ii) found that a significant reduction in SF over time occurred for the un-sheared sample, with a reduction from 8.0 to 1.9 over 11 days (Fig. 5a). With such significant loss in SF over time without any shearing, it is perhaps unsurprising that no recovery was observed after both shearing treatments (Fig. S21†). This aqueous solution instability of T1 Fe(ii) is likely, at least in part, due to the presence of a hydrolysable ester linking group between the main-chain and terpyridine end-functionality. To highlight this point, the solution stability of T1 Fe(ii) was compared with T3 Fe(ii), which does not contain an ester linking group (Fig. 5a). Some reduction in SF over time for solutions of T3 Fe(ii) was also observed (4.8 to 3.4 over 10 days), however this was at a significantly reduced rate than for T1 Fe(ii). The cause of SF reduction in the case of T3 Fe(ii) was not determined, however gradual hydrolysis of the central trithiocarbonate group is a likely candidate.

Shear recovery experiments for T3 Fe(ii) were then conducted and both shearing treatments resulted in a large initial reduction in SF from 4.5 to 2.1 (5000 RPM, 15 min) and 4.5 to 2.3 (3200 RPM, 60 min). This was then followed by a slow recovery over time, which peaked at an SF of approximately 3.0 for both treatments after 5 days, with the majority of this recovery occurring within the first 24 hours (Fig. 5b). While complete recovery is certainly not observed, the difference in SF between un-sheared and sheared states after 10 days is less than 0.5 (recovery to 88% of un-sheared SF, Fig. S22a†), indicating considerable recovery from polymer chain scission due to shearing. The incomplete nature of recovery is proposed to be due to the occurrence of both coordination and covalent bond scission. With a disperse high molecular weight telechelic base polymer (*M*_n,GPC_ = 551 kDa, *Đ* = 1.28), the presence of some telechelic species large enough to undergo covalent bond scission during shearing is possible.

These results also provide some insight into the mechanism of shear recovery for UHMW terpyridine–Fe(ii) LCPs. Given that increases in SF were observed up to 5 days after mechanical shearing and that only 1 hour is needed to form the Fe(ii) LCP from an un-complexed polymer, recovery through re-association of completely dissociated ligands is unlikely to be the only pathway and may not contribute at all. Therefore, loss in molecular weight after shearing may be due to the formation of cyclic species rather than an increased presence of un-complexed ligand, as was proposed by Sijbesma *et al.* when investigating smaller LCPs after sonication induced coordination bond scission.^25^ In this case, recovery to equilibrium molecular weight occurs through ligand exchange interactions, which fits well with our data since the ligand exchange half-life of the Fe(ii)–terpyridine complex is relatively long (140 hours at 35.4 °C). By using this insight, the rate of recovery was improved by trialling the Ni(ii) complex, which has a ligand exchange half-life approximately 4.5 times shorter (10.2 hours at 44.8 °C).

Preliminary data indicated that 24 hours was needed for the Ni(ii) complex to fully form in dilute solution (likely due to its lower formation rate constants) and therefore the shearing was applied 24 hours after Ni(ii) addition to allow for full complex formation (Fig. 5c). For the un-sheared sample, a reduction in SF from 4.3 (at 24 hours) to 3.5 (8 days) was observed, similar to the rate of reduction for the un-sheared Fe(ii) complexed polymer. After shearing, a noticeably accelerated rate of recovery was observed (compared to the Fe(ii) sample), with SF increasing from 2.0 to 3.4 after 24 hours from treatment at 5000 RPM (15 minutes). In the same amount of time, the Fe(ii) sample SF only recovered from 2.1 to 2.6 and eventually reached 3.0 after 5 days. Similar to the Fe(ii) complex, a peak recovery up to 91% of un-sheared SF was observed (Fig. S22b†). With the Ni(ii) complex having a slower association rate, but quicker ligand exchange rate, this data further supports the theory that recovery from mechanical shear for UHMW LCPs is governed by ligand exchange interactions.

To get a picture for how these UHMW LCPs compare to the current covalent polymers after shearing, the data for AM-5-6M before and after shearing at 5000 RPM for 15 minutes (100 ppm) is presented in Fig. 5d. This was then compared to both T3 Fe(ii) and T3 Ni(ii) before and after (point of greatest recovery) shearing. This demonstrates that (when given enough time) UHMW LCPs can outperform traditional UHMW covalent polymers in terms of minimising SF reduction after shear treatment. On the way to seeing the developed UHMW LCPs applied in spray drift reduction, further investigation into the cause of SF reduction over time in water will be needed, as well as the testing of these polymers in active solutions (containing other salts/organic compounds).

## Conclusion

This report details the synthesis, testing and optimisation of UHMW LCPs (*M*_n_ > 1000 kDa) containing the terpyridine ligand. Telechelic polymers of approximately 400 kDa were synthesised by RAFT polymerisation of AM with bis-terpyridine end-functionalised macro-CTAs. The terpyridine end-functional polymers associated into UHMW LCPs after Fe(ii) complexation, as determined by SF, MALS GPC and intrinsic viscosity measurements. In addition, the UHMW LCPs were found to be effective DCAs, reducing the formation of fine driftable droplets (those <105 μm in diameter) during spray testing at concentrations as low as 100 ppm. These LCPs were also shown to recover from mechanically induced coordination bond scission, with the rate of partial (up to 90% of an un-sheared sample after the same time in solution) SF recovery found to be dependent on the transition metal ion. The Fe(ii) complex recovered over approximately 4–5 days, while the Ni(ii) complex required only 24 hours.

## Conflicts of interest

There are no conflicts of interest to declare.

## Supplementary Material

Supplementary information
