# Supplementary material for: Ultra-high molecular weight linear coordination polymers with terpyridine ligands
Source: Chem Sci. 2019 May 15;10(24):6174–83. doi: 10.1039/c9sc01115c (PMC6585884; doi:10.1039/c9sc01115c)
Supplement: Supplementary file 1 [file SC-010-C9SC01115C-s001.pdf]

Electronic Supplementary Material (ESI) for Chemical Science.
This journal is © The Royal Society of Chemistry 2019

Supporting Information

Ultra-High Molecular Weight Linear Coordination Polymers with Terpyridine Ligands

Reece W. Lewis[a], Nino Malic[b], Kei Saito[c], Richard A. Evans[b*], Neil R. Cameron[a,d*]

[a]Department of Materials Science and Engineering, Monash University, 22 Alliance Lane, Clayton, Victoria, 3800, Australia

[b]CSIRO Manufacturing Flagship, Clayton, 3168, Australia

[c]School of Chemistry, Monash University, Clayton, 3800, Australia

[d]School of Engineering, University of Warwick, Coventry, CV4 7AL, UK

Table of Contents

SUPPORTING DATA ITEMS 3

STABILITY CONSTANT $M_{N,TH}$ CALCULATIONS 3
MACRO-CTA SYNTHESIS 6
INITIAL CHAIN EXTENSIONS OF M1 10
EXTINCTION COEFFICIENT EVALUATION 13
COORDINATION COMPLEXES OF T2 WITH OTHER METALS 14
HIGH MOLECULAR WEIGHT TELECHELICS 17
CONTROL ADJUVANT SPRAY DROPLET SIZE ANALYSIS 18
POLYMER / SHEAR STABILITY 21

EXPERIMENTAL PROCEDURES 23

MATERIALS 23
RAFT AGENT SYNTHESIS 23
POLYMER SYNTHESIS 35
COORDINATION POLYMER SYNTHESIS 37
NMR SPECTROSCOPY 39
UV-VIS SPECTROSCOPY 39
VISIBLE LIGHT SOURCES 39
GEL PERMEATION CHROMATOGRAPHY 41
VISCOSITY MEASUREMENTS 42
SCREEN FACTOR WATER MEASUREMENTS 43
DROPLET SIZE MEASUREMENTS 44

SUPPORTING REFERENCES 45

Supporting Data Items

Stability constant $M_{n,th}$ calculations

The formation of LCPs from bi-functional monomers by 1:2 metal/ligand complexation (as is the case for terpyridine based LCPs) is displayed in **Scheme S1**. This system can be described by the 4 simultaneous equations below:

$$[ML] = K_1[M][L]$$

$$[ML_2] = K_2[ML][L]$$

$$0 = [L_0] - [L] - [ML] - 2[ML_2]$$

$$0 = [M_0] - [M] - [ML] - [ML_2]$$

After substituting known values for K1, K2, $[L_0]$ and $[M_0]$ for a given situation, computational simultaneous solving of the above equations for [M], [L], [ML], $[ML_2]$ gives the theoretical distribution of species at equilibrium. This can be done by a four stage iterative process in excel.

1. Solve for $[M]_y$ ([M] at iteration y) by solving the equation below. In the first iteration set $[M]_{y-1}$ to 0 and set $[ML_2]_{solver}$ to the output optimised by excels solver function.

$$[M]_y = \frac{[M_0] - [ML_2]_{solver}}{(1 + K_1([L_0] + [M]_{y-1} - [M_0] - [ML_2])}$$

2. Use the output of $[M]_y$ in the following equations to evaluate [L], [ML] and $[ML_2]_{check}$.

$$[L] = [L_0] + [M] - [M_0] - [ML_2]$$

$$[ML] = [M_0] - [M] - [ML_2]$$

$$[ML_2]_{check} = K_1K_2[M]([L_0] + [M] - [M_0] - [ML_2])^2$$

3. Create a cell with the following equation to be optimised to 0 in excels solver function. Run the solver program to set this value to 0 by changing variable cell $[ML_2]_{solver}$

$$Solver\ 0\ cell = [ML_2]_{check} - [ML_2]_{solver}$$

4. Once a solution for $[ML_2]_{solver}$ is converged upon, re-iterate the above 3 equations until $[M]_y \approx [M]_{y-1}$

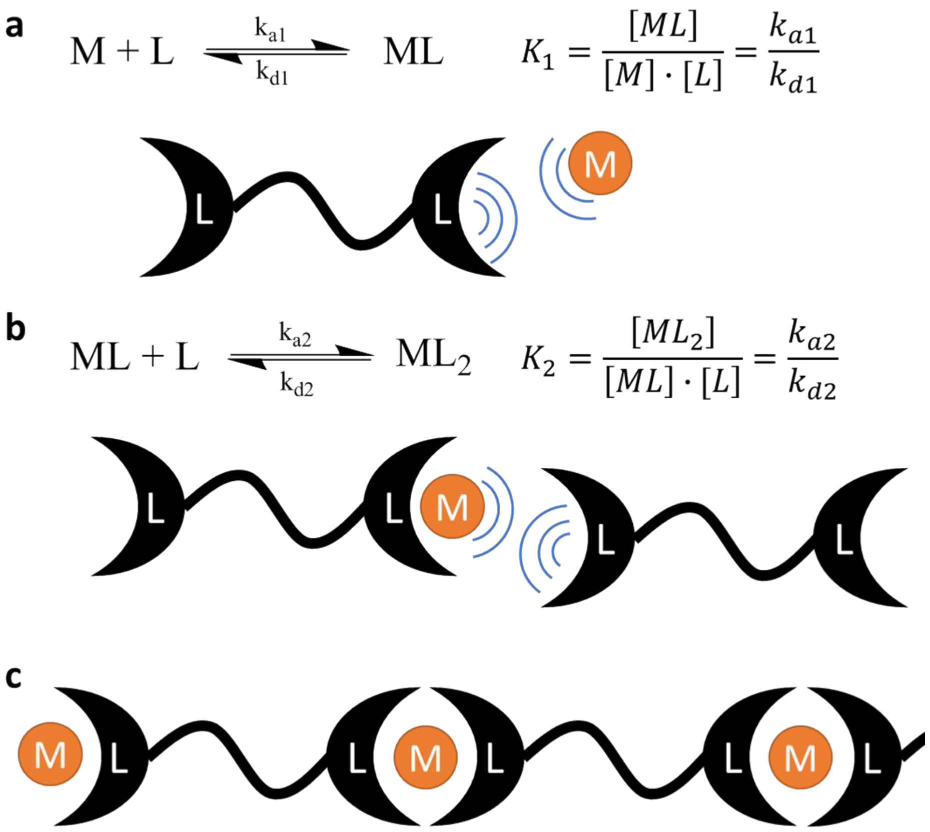

Scheme S1. Simplified reaction scheme for the formation of LCPs from 1:2 metal/ligand complexation, related to Table 1. a) Equilibrium for formation of the mono-complex, b) equilibrium for the formation of the bis-complex and c) schematic structure of formed LCP. Square brackets denote concentration. k_{a1} and k_{a2} represent the association rate constant for steps one and two respectively, while k_{d1} and k_{d2} represent the analogous dissociation rate constants.

Once the value for $[ML_2]$ has been determined, the DP can be evaluated from the following rearrangement of the Carothers equation where ρ is conversion of monomer to supramolecular species and f_{av} is the functionality of the monomeric species.[1] In the case of 100% end-group fidelity f_{av} is 2 for the bi-functional monomers used in LCP synthesis. This equation can then be rearranged to give the DP in terms of $[ML_2]$, $[L_0]$ and EGF (end-group fidelity fraction). Lastly $M_{n,th}$ can be calculated by multiplying the DP by the molecular weight of the bi-functional monomer (M_M).

$$DP = 2/(2 - \rho f_{av})$$

$$DP = \frac{1}{1 - (\frac{2 \times [ML_2]}{[L]_0}) \times EGF}$$

$$M_{n,th} = DP \times M_M$$

These equations were then used to model the (maximum) theoretical molecular weights formed from association of 400 kDa bis-terpyridine functionalised monomers at 1 mg/mL (**Table S1**).

Table S1. Computational simulation results for terpyridine LCPs from 400 kDa monomers bi-functional monomers at 1 mg/mL, related to Table 1.

Complex	K_1 M^{-1} (Log_{10})	K_1 M^{-1} (Log_{10})	[M] M x10^{-10}	[L] M x10^{-10}	[ML] M x10^{-10}	[ML_2] M x10^{-10}	DP (EGF = 1.00)	DP (EGF = 0.95)
**Fe(II) tpy**	7.1	13.8	0.91	1.82	0.002	24.1	27.4	11.8
**Ni(II) tpy**	10.7	11.1	0.16	1.54	1.22	23.6	18.1	9.77
**Co(II) tpy**	9.5	9.1	2.12	12.9	8.70	14.2	2.31	2.17
**Cu(II) tpy**	12.3	6.8	0.005	24.6	24.6	0.38	1.02	1.01
**Zn(II) tpy**	8.2	6.1	15.2	40.2	9.71	0.05	1.00	1.00

Macro-CTA synthesis

Figures S1 - S3 show the ^{1}H NMR spectroscopic characterisation data for the macro-CTAs M1 – M3, as well as an example of a low α-proton integral macro-CTA which was shown to ultimately produce low end-group fidelity polymers (M4). M4 was synthesised by polymerising DMA with tpyTTC in THF without temperature control (allowing the polymerisation exotherm to heat the polymerisation) and resulted in an α-proton integral of 1.13, well below the theoretical 2 (**Table S2**). Chain extension of M4 with AM (T4, $M_{n,conv}$ = 23.6 kDa) was found to qualitatively result in a polymer with retention of terpyridine end-groups (as demonstrated by UV-Vis spectroscopy on the Fe(II) complex), however little increase in relative viscosity was observed after Fe(II) (and other metal) complexation and was attributed to the presence of many mono-telechelic type polymers (**Figure S5**). It should be noted that by GPC the polymerisation resulting in M4 did appear to be well controlled, with a low dispersity (□ = 1.09) and reasonable agreement between $M_{n,GPC}$ (5.61 kDa) and $M_{n,conv}$ (6.48 kDa). This highlights the utility of using the α-proton integral in quality control. A proposed general structure for the impurities present M4 resulting in a reduced α-proton integral is presented in **Figure S4**.

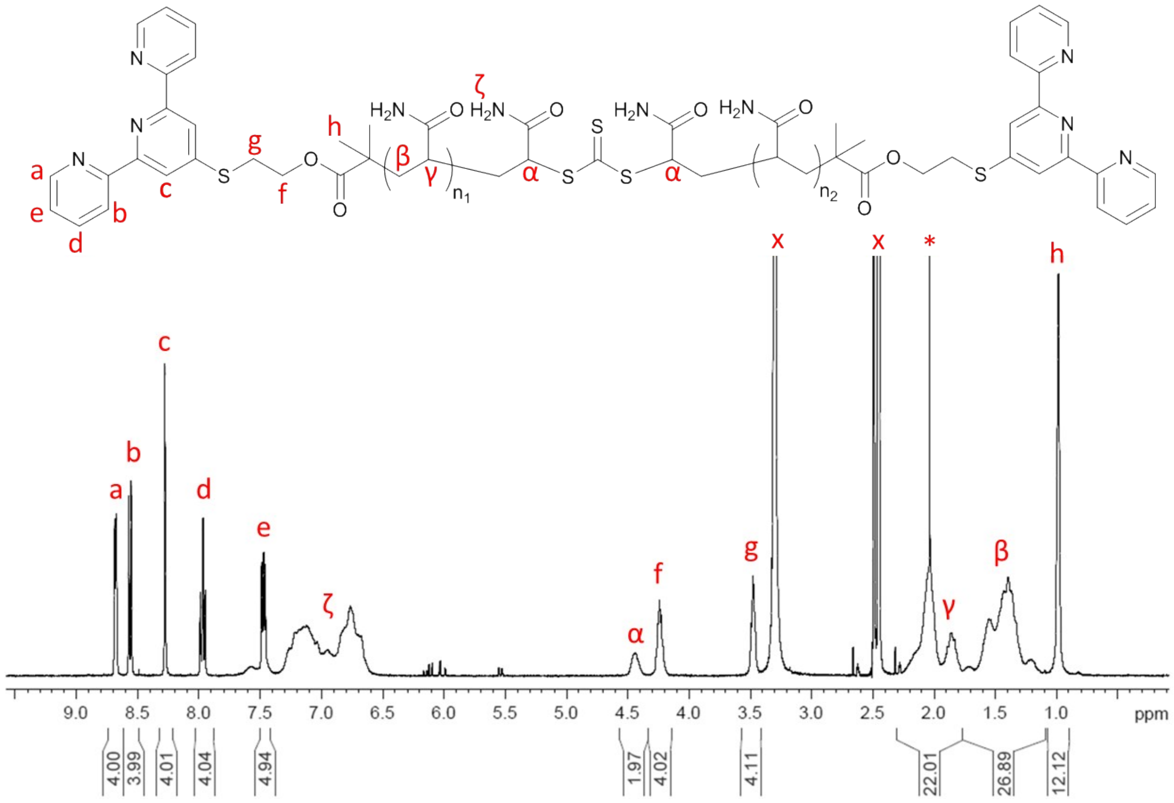

Figure S1. Chemical structure and ^{1}H NMR spectrum (400 MHz, DMSO-d_6) of M1. *Residual acetone signal.

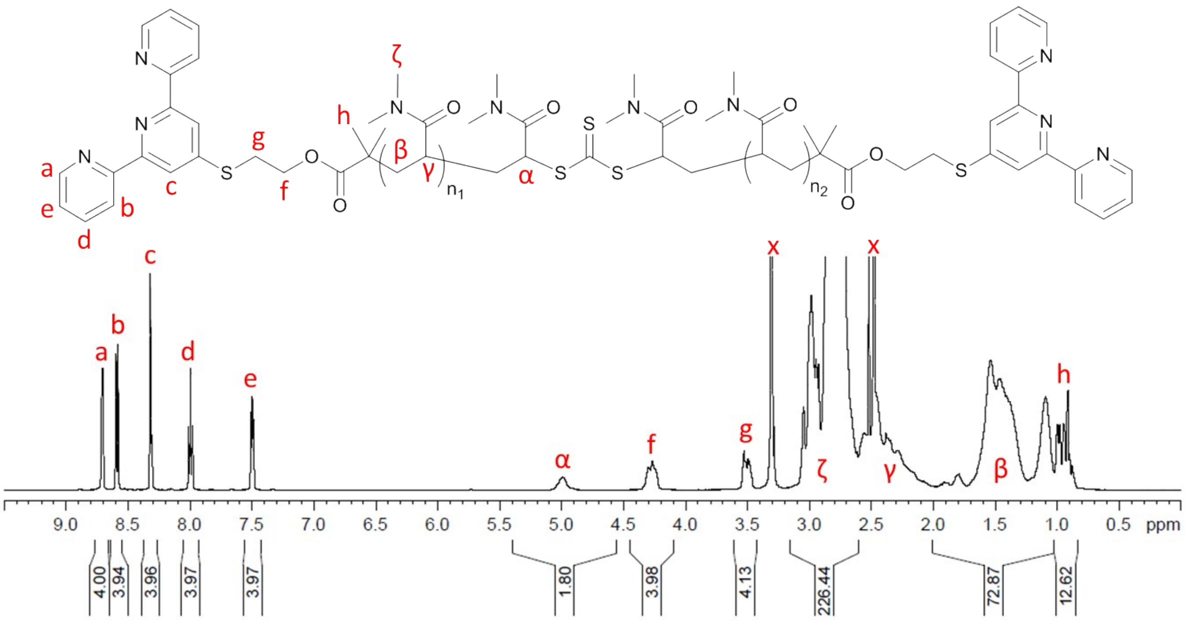

Figure S2. Chemical structure and ^1H NMR (500 MHz, DMSO-d_6) spectrum of M2, related to data in Table 2.

Table S2. Polymerisation conditions and characterisation data for polymers not presented in main article.

Code	CTA	[monomer] : [CTA]	Polym time (h)	Light power	Conv (%)	$M_{n,conv}$ (kDa)	$M_{n,NMR}$ (kDa)	α-proton integral
**M4**[a]	tpyTTC	70 (DMA)	0.6	52 W	73	6.48	7.82	1.13
**T4**[b]	M4	235 (AM)	5.5	12 W	94	23.6	33.4	-
**T5**[c]	M1	235 (AM)	4.0	104 W	89	16.0	16.2	-

[a]Macro-CTA was synthesised in THF, under 402 nm irradiation. [b]$M_{n,UV}$(T4) = 27.4 ± 0.7 kDa [c]$M_{n,UV}$(T5) = 18.3 ± 1.9 kDa

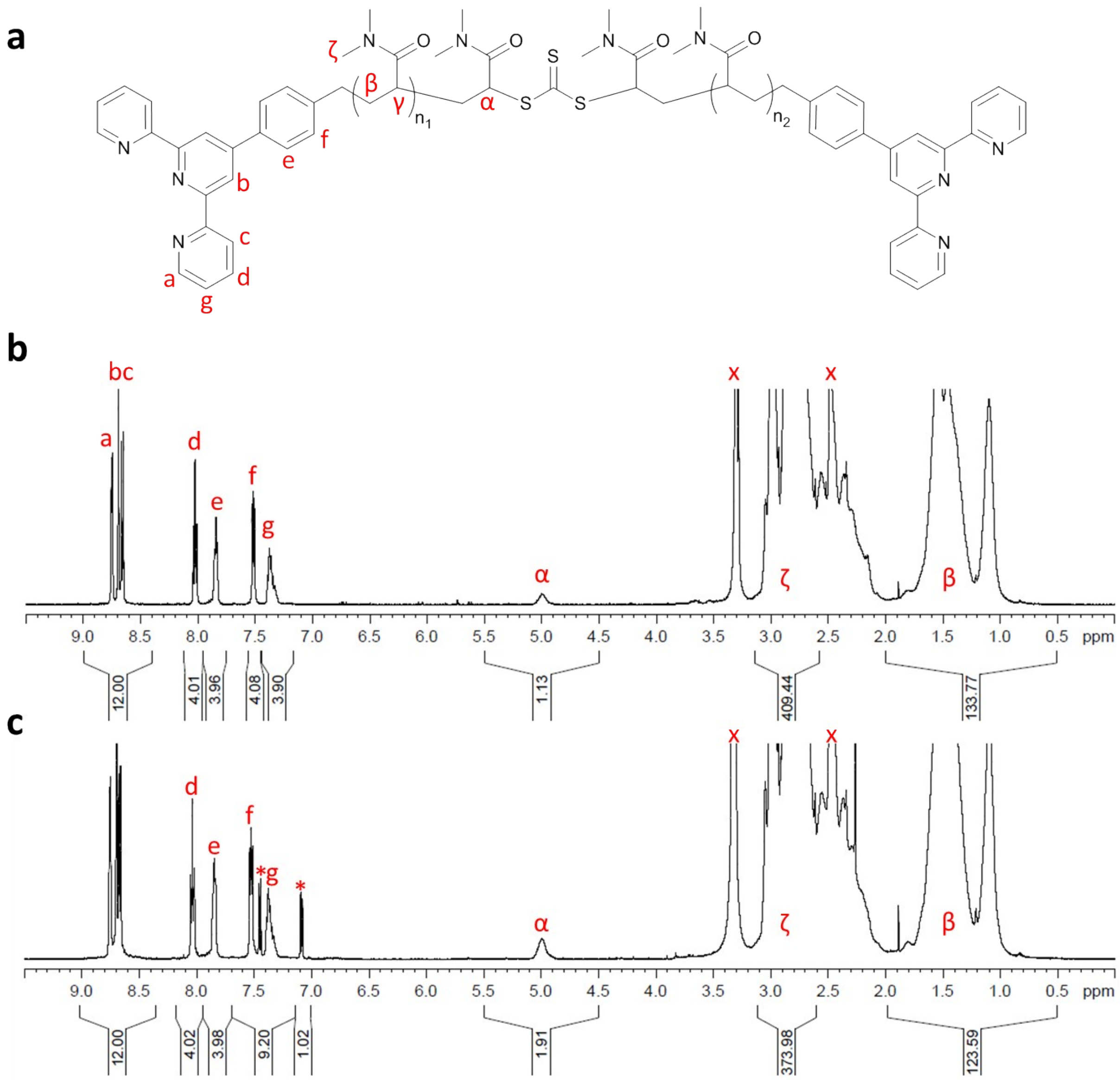

Figure S3. a) General chemical structure of DMA tpyTTC macro-CTAs (M3 and M4), related to data in Table 2. ^{1}H NMR (500 MHz, DMSO-d_6) spectrum of b) M4 and c) M3. *Residual PTSA.

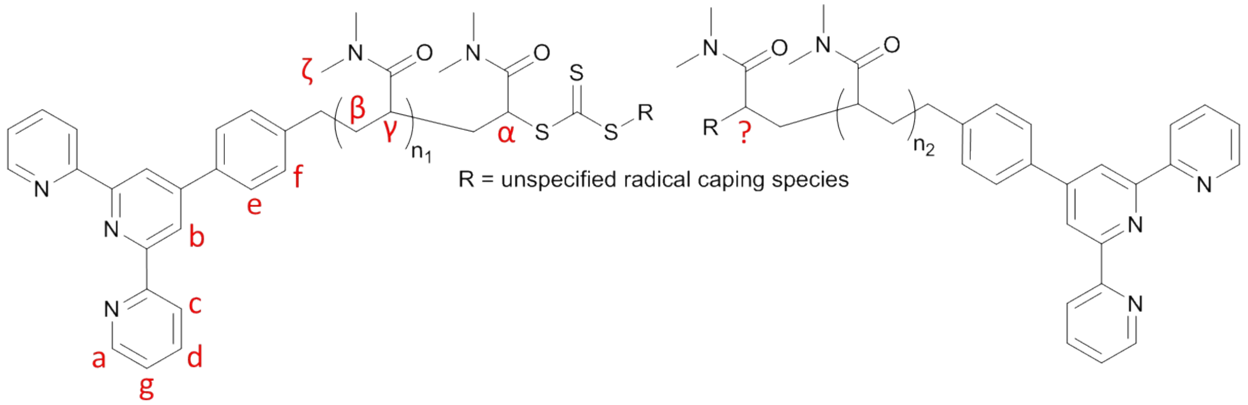

Figure S4. Proposed chemical structure of impurities in M4.

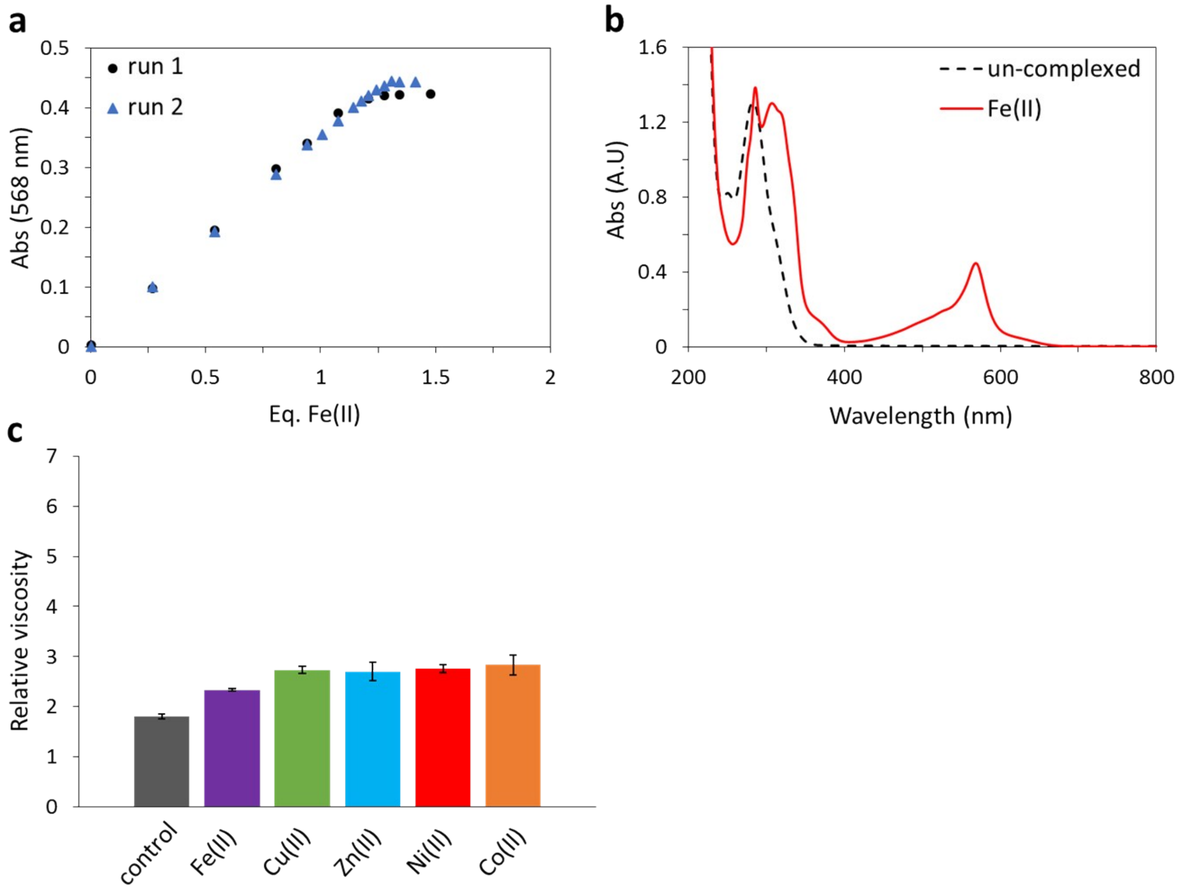

Figure S5. Fe(II) titration of T4 demonstrating the formation of polymeric species with significant retention of terpyridine functionality (a – b). c) Relative viscosity data from T4 complexed with various transition metal ions showing little increase in molecular weight with complexation, suggesting the presence of many mono-telechelic type species stopping chain growth.

Initial chain extensions of M1

During preliminary experiments the macro-CTA M1 was chain-extended with AM to approximately 15 kDa (relatively low molecular weight to allow for simplified characterisation), resulting in the telechelic polymer T5 (**Table S2**). Overall the polymerisation appeared to be well-controlled, with (after a short induction period) linear pseudo-first order kinetics observed, indicating that the concentration of active species was constant (**Figure S6a**). In addition, $M_{n,GPC}$ increased with conversion, while dispersities remained low throughout (□ < 1.15, **Figure S6b**). The slight deviation from a linear increase in $M_{n,GPC}$ with conversion may be due to terpyridine end-group interactions with the column, particularly at lower molecular weights. Indeed, initial attempts to monitor the polymerisation with a different aqueous GPC system resulted in what appeared to be the polymer eluting after the solvent, along with other unusual artefacts strongly suggesting the presence of deleterious column interactions (**Figure S7**). By comparing the integrals of the polymer backbone to the terpyridine end-groups, an $M_{n,NMR}$ of 16.2 kDa was evaluated for T5, in close agreement with $M_{n,conv}$ (16.0 kDa).

Solutions the of T5 (0.05 mM) were then titrated against increasing equivalents of Fe(II) (equivalents based on $M_{n,NMR}$) (**Figure S6c**). A linearly increasing absorbance at 566 nm was observed, which then reached a peak close to the calculated equivalence of Fe(II) from $M_{n,NMR}$ and then remained constant. Viscometric analysis was then conducted to confirm the formation of LCPs upon complexation of T5 with Fe(II). After complexation, a 380% increase in relative viscosity at 20 mg/mL was observed, indicating the formation of higher molecular weight species (**Figure S6d**).

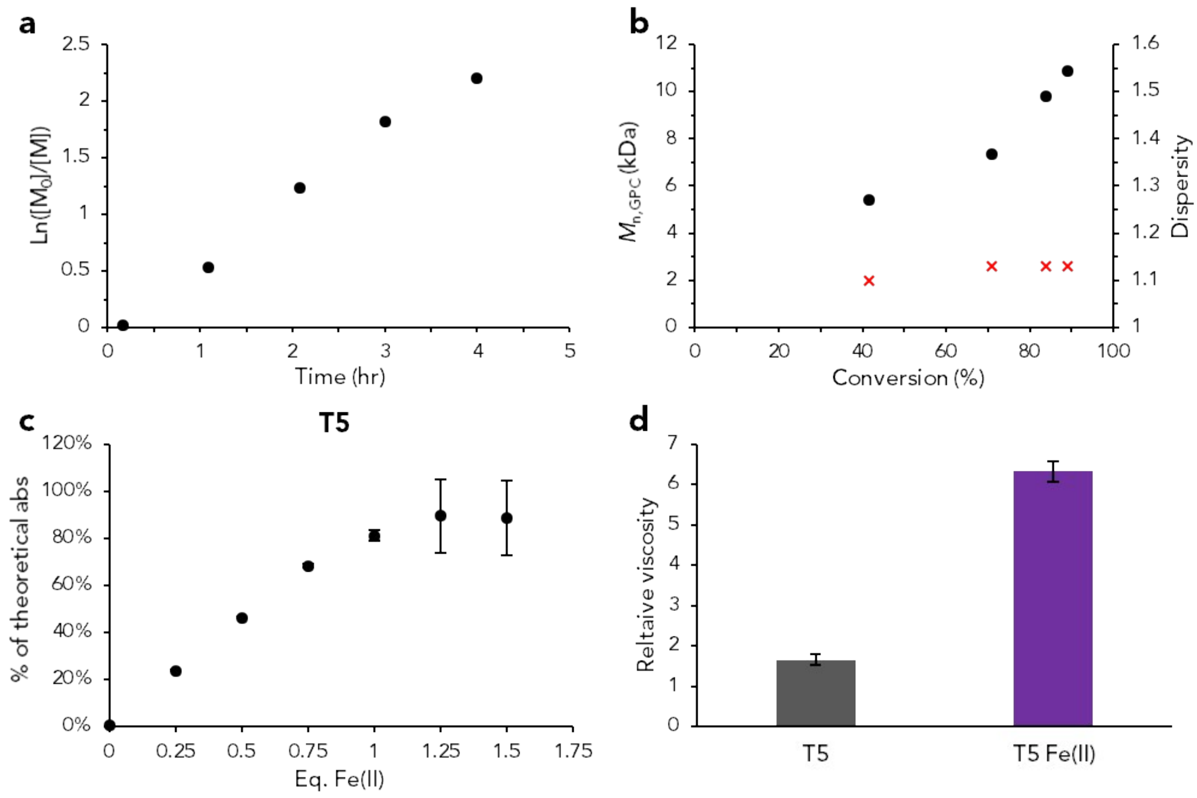

Figure S6. Characterisation data for DP-RAFT polymerisation of AM with tpyDMAT macro-CTA M1 ([M1]:[AM] = 234) resulting in the polymer T5. (a) Kinetic plot demonstrating linear pseudo-first order kinetics and (b) $M_{n,GPC}$ and ▫ plotted against conversion (data from the Tosoh GPC system) demonstrating growth of polymer chain with increasing conversion. (c) Absorbance at 566 nm for aqueous solutions of T5 in the presence of increasing equivalents of Fe(II), expressed as a percentage of theoretically calculated fully complexed absorbance from $M_{n,NMR}$ and ε_{566} = 9536 $cm^{-1}M^{-1}$ (values are the average of two independent measurements ± STD). (d) Relative viscosity data (20 mg/mL) for un-complexed and Fe(II) complexed T5 (values are average of two independent measurements ± STD).

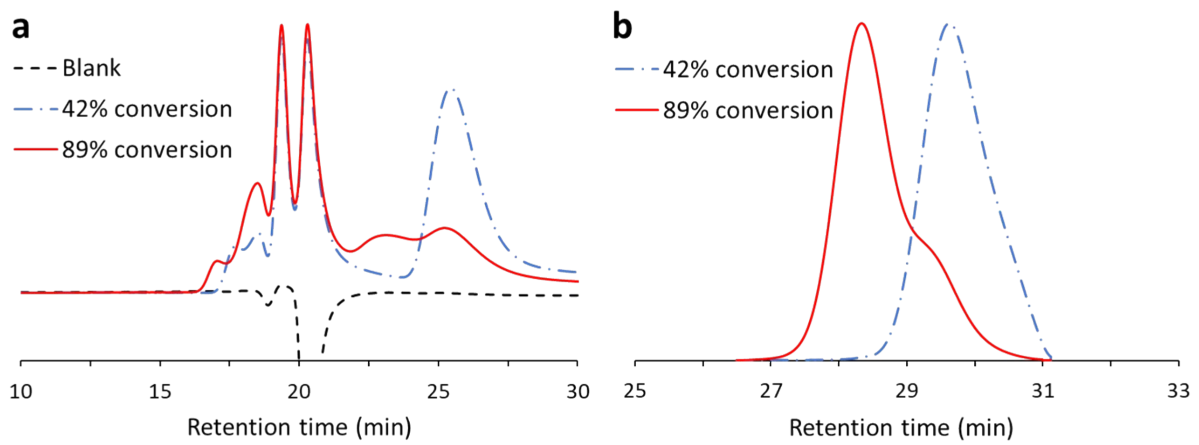

Figure S7. a) Evidence of column interactions for low molecular weight terpyridine telechelic polymers on the Waters aqueous GPC system, with non-standard RI traces appearing around or after the solvent peak during synthesis of T5. b) Matching samples analysed on the Tosoh GPC system.

Extinction coefficient evaluation

Standards of tpy-OH were used to determine the extinction coefficient to closely match the chemistry of the ligands attached to T1 and T2. While tpy-OH is insoluble in water, its Fe(II) complex is soluble, allowing for aqueous solution UV-Vis absorbance measurements. Aqueous solutions of three different concentrations were analysed, with independently prepared repeats at each concentration. A standard deviation of less than 0.004 AU was recorded across the repeats at all concentrations, with the average absorbance at each concentration presented in **Figure S8**. By linearly fitting the data, a gradient of 9.536 was evaluated, resulting in ε_{566} = 9536 $cm^{-1}M^{-1}$ for the tpy-OH Fe(II) complex. Note that the predominant species in solution is expected to be the bis-terpyridine Fe(II) complex due to its significantly higher stability constant compared to the mono-complex.

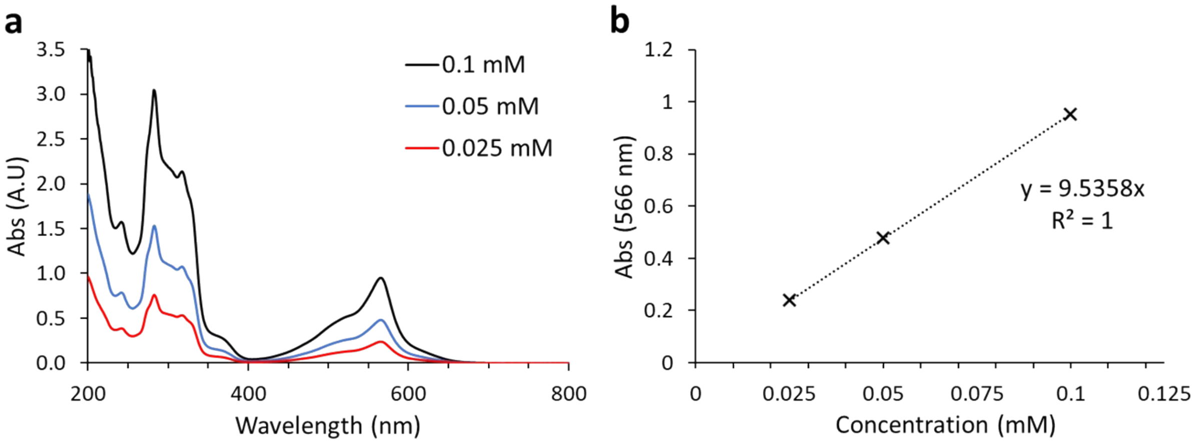

Figure S8. UV-Vis absorbance calibration for the tpy-Fe(II) complex utilising tpy-OH as a model compound in aqueous solution. a) Full UV-Vis absorbance spectrum for different concentrations of tpy-OH Fe(II), b) change in absorbance at 566 nm with increasing concentration of tpy-OH Fe(II).

In order to evaluate $M_{n,UV}$ for T3 and T4, a new extinction coefficient was required as the terpyridine ligand structure of tpyTTC is different to tpyDMAT (with which the original calibration was obtained). This difference is highlighted in **Figure S9**, demonstrating the different Fe(II) complex absorption for T4 and T5 (lower molecular weight species compared to increase fidelity of measurement). The new extinction coefficient was evaluated by fitting the linear region of the terpyridine polymer Fe(II) titration curves on the assumption that only bis-complexes were formed. To test this method, the calculation was first performed for T5 (made from tpyDMAT) and an extinction coefficient of 8760 $cm^{-1}M^{-1}$ resulted (**Figure S10a**). This is within 10% of the originally calculated value from tpy-OH (9536

cm^{-1}M^{-1}), demonstrating the applicability of this method. Performing the calculation on the T4 titration yields an extinction coefficient of ε_{568} = 11 740 cm^{-1}M^{-1} (**Figure S10b**).

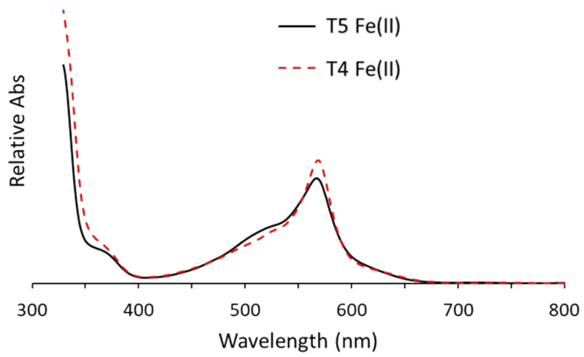

Figure S9. Comparison of the absorbance spectra for terpyridine polymers complexed with Fe(II) made from tpyDMAT (T5 Fe(II)) and tpyTTC (T4 Fe(II)).

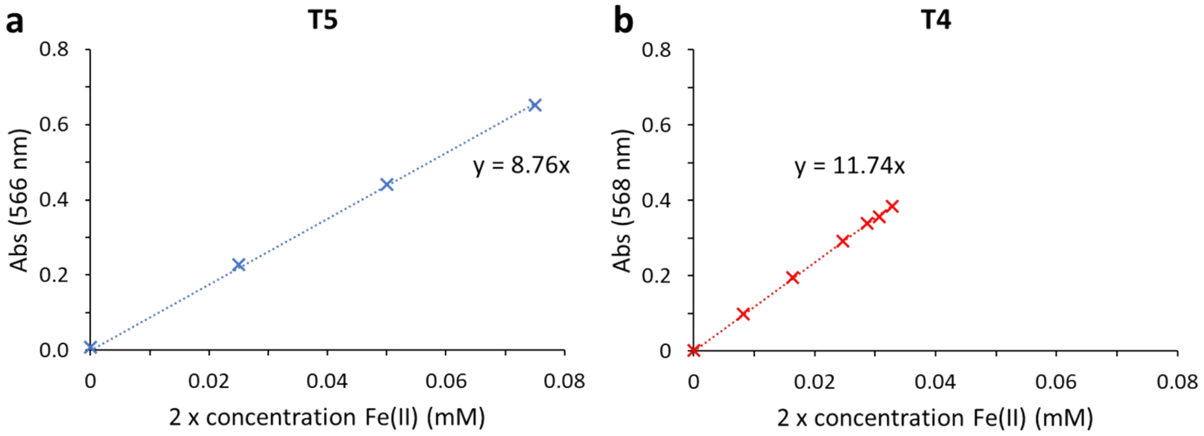

Figure S10. Fitted absorbance data for terpyridine end-functional polymers (made from: a) tpyDMAT and b) tpyTTC) titrated with Fe(II) as a function of 2 times the concentration of Fe(II) in solution (theoretical concentration of bis-complexed terpyridine).

Coordination complexes of T2 with other metals

A library of LCPs from T2 complexed with different metal ions was synthesised, with the metal ions added at two different polymer concentrations (10c = 10 mg/mL, 2c = 2 mg/mL). A lower polymer

concentration during metal ion addition (2 mg/mL) was implemented to explore if UHMW LCPs could form under equilibrium conditions at such dilutions (rather than diluting after formation at 10 mg/mL and potentially analysing out of equilibrium). The molecular weight of these LCPs was then characterised by relative viscosity, aqueous GPC and SF measurements (**Figure S11**).

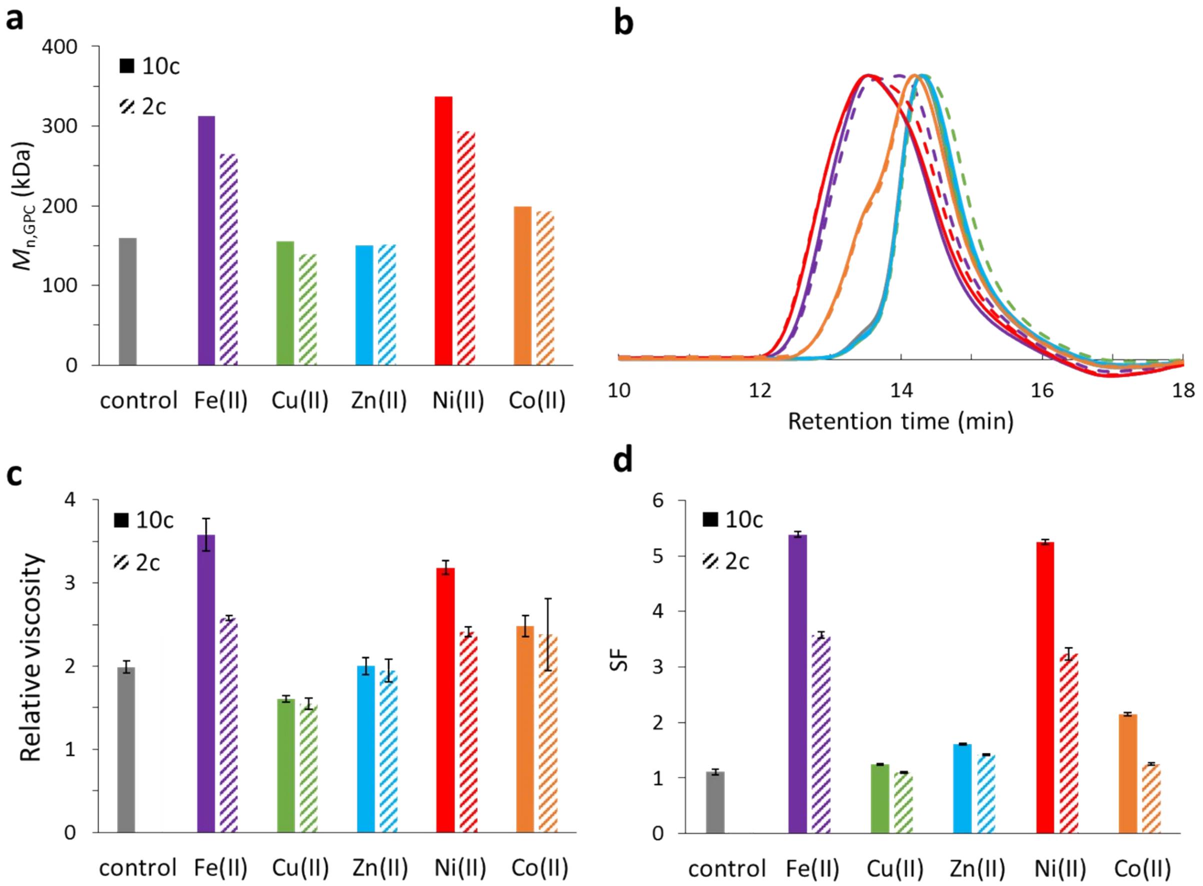

Figure S11. Effect of different metal ion complexes on molecular weight of T2 LCPs as measured by a) $M_{n,GPC}$ (1 mg/mL), b) GPC traces (solid line = 10c, dashed = 2c, trace colours as per rest of figure), c) relative viscosity (2 mg/mL) and d) Screen Factor (1 mg/mL). 10c corresponds to samples complexed at 10 mg/mL and 2c corresponds to samples complexed at 2 mg/mL.

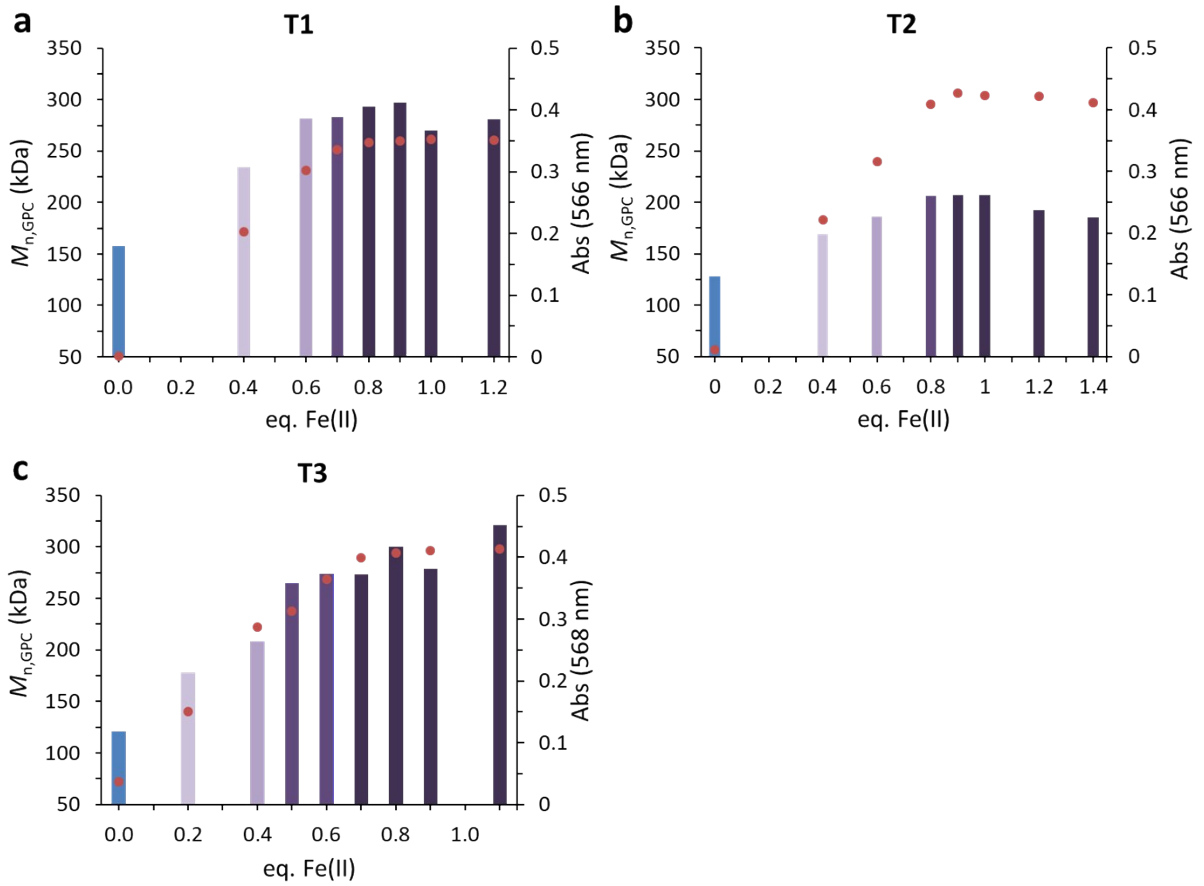

Figure S12. GPC characterisation data (Waters system, PEO calibrated) for Fe(II) LCPs synthesised from the high molecular weight telechelics T1 – T3, relates to data in Figure 2.

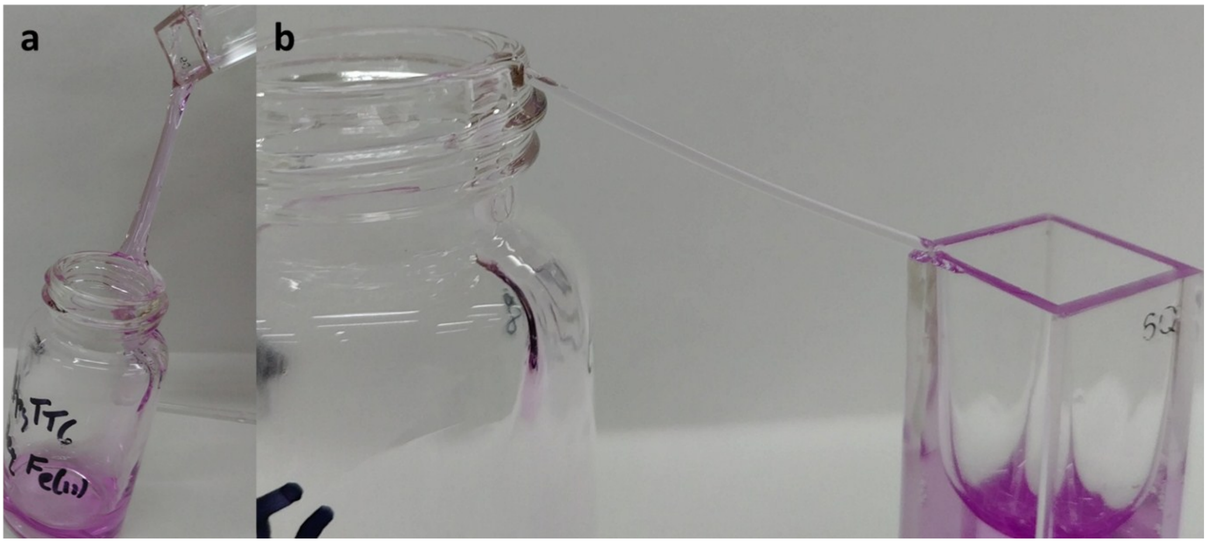

Figure S13. Demonstration of high extensional viscosities encountered during formation of T3 Fe(II) at 10 mg/mL. a) Stable thick filament formed when transferring T3 Fe(II) for UV-Vis analysis, b) stable filament of T3 Fe(II) bridging vial and UV-Vis cuvette.

High molecular weight telechelics

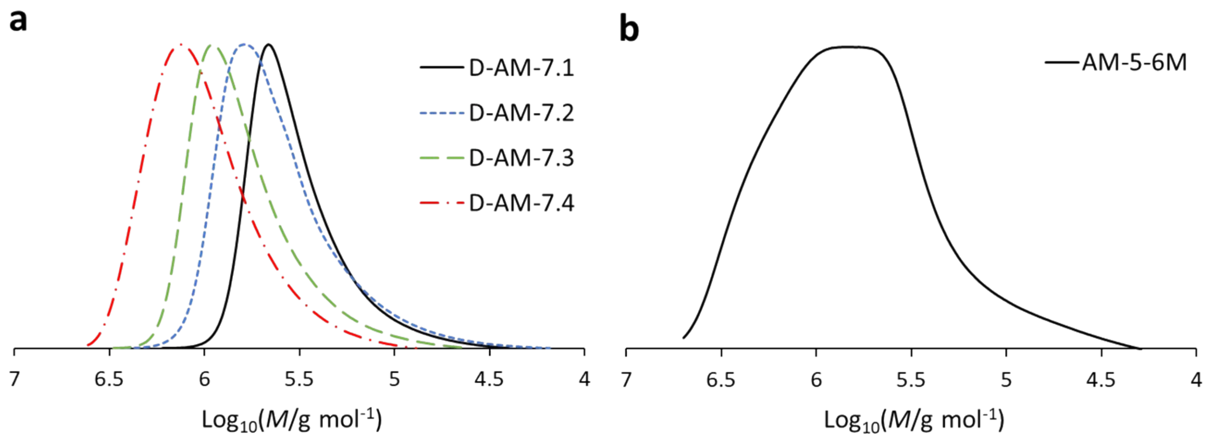

Figure S14. Aqueous GPC characterisation (Waters system) of a) control carboxylic acid end-functional polymers and b) commercial free radical polyacrylamide (AM-5-6M). Relates to data in Table 4.

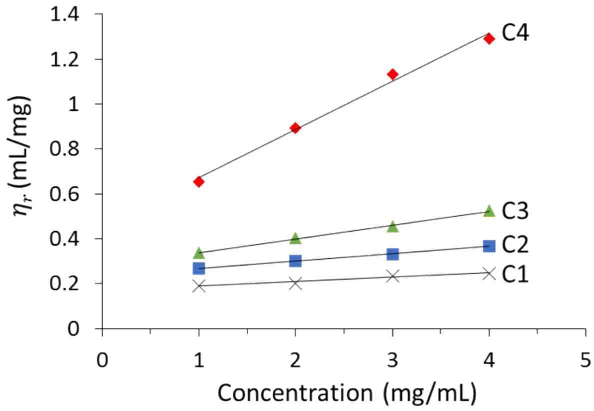

Figure S15. Full reduced viscosity data for control polymers (C1 – C4, main results presented in Figure 3).

Table S3. Intrinsic viscosity and calculated molecular weight of LCP and control polymers, relates to data in Figure 3.

Code	Intrinsic viscosity (mL/mg)	$M_{n,conv}$ (kDa)	$M_{w,visc}$ (kDa)
**AM-5-6M**	1.27	-	5750
**T1 Fe(II)**	0.74	-	2180
**C4**	0.46	2747	1480
**C3**	0.28	1519	763
**C2**	0.23	807	611
**C1**	0.17	444	397

Control adjuvant spray droplet size analysis

An extended data set of the droplet size analysis presented in the main work has also been presented in terms of Dv_{10}, Dv_{50} and Dv_{90}. An example cumulative volume distribution (for water only) has been presented below, with the key distribution measures marked (**Figure S16**). Dv_{10} for example corresponds to the droplet diameter value for which 10 volume percent of the spray is smaller than. Dv_{50} and Dv_{90} then correspond to the diameters for which 50 and 90 volume percent of the spray is smaller than, respectively.

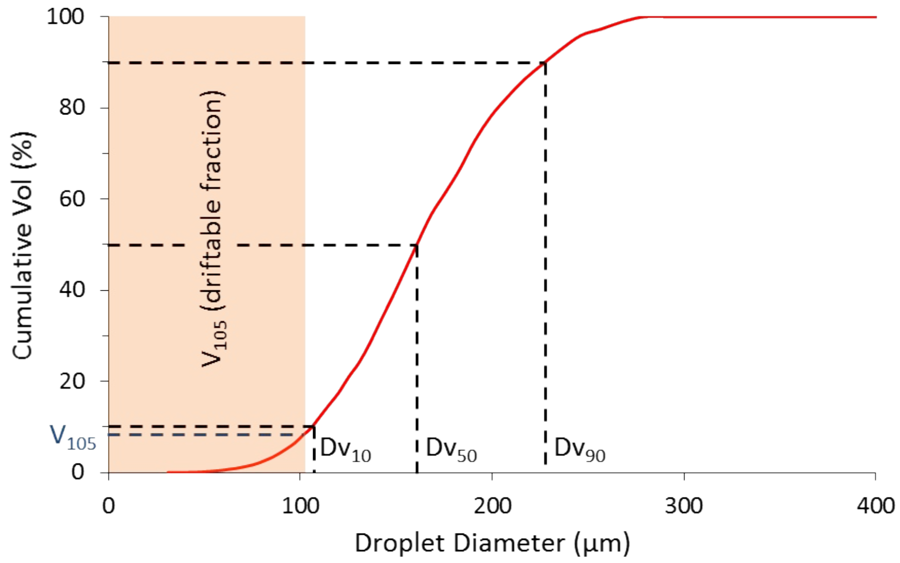

Figure S16. Example cumulative volume spray distribution with Dv_{10}, Dv_{50}, Dv_{90} and V_{105} marked.

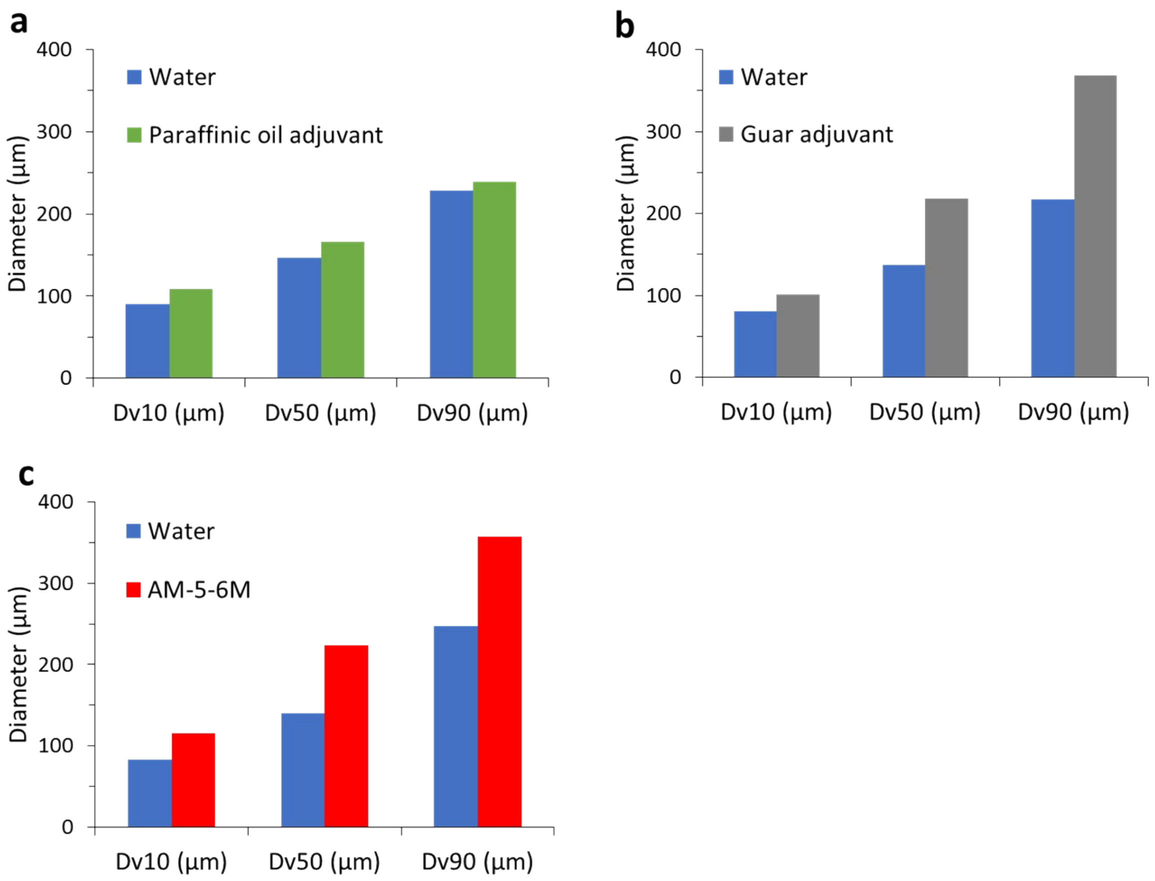

Figure S17. Additional spray droplet size analysis for commercial DCAs, relates to data in Figure 4. a) Paraffinic oil adjuvant at 0.5 volume %, b) guar adjuvant at 0.5 volume % and c) AM-5-6M at 100 ppm.

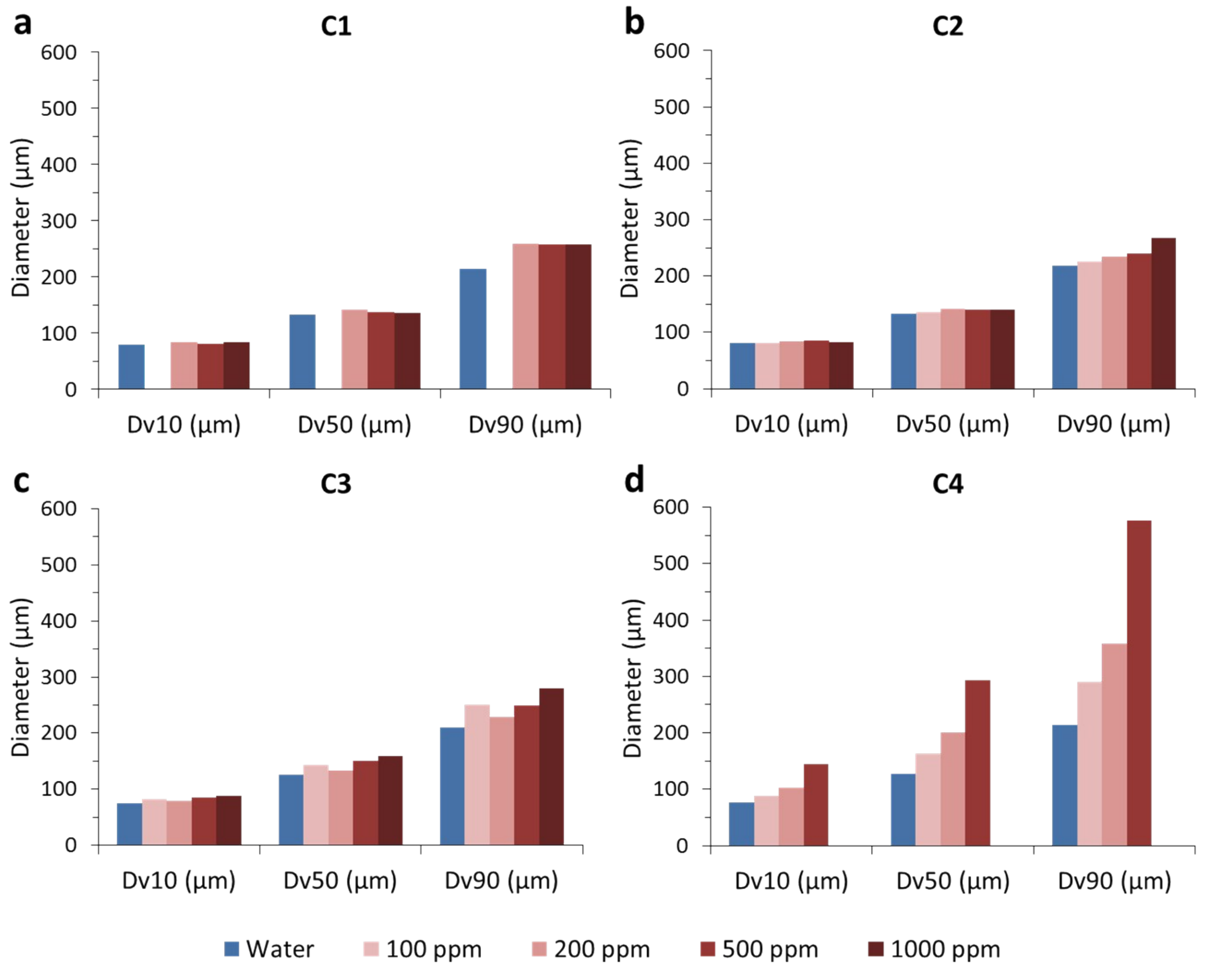

Figure S18. Additional spray droplet size analysis for control polymer samples C1 – C4 analysed at different concentrations (100 – 1000 ppm), relates to data in Figure 4.

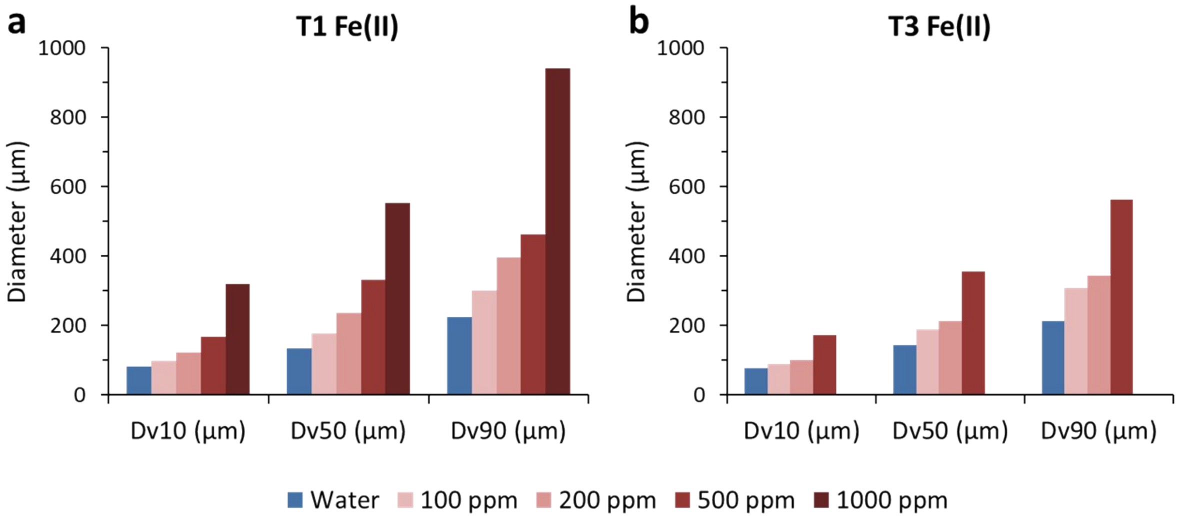

Figure S19. Additional spray droplet size analysis for LCPs T1 Fe(II) and T3 Fe(II) analysed at different concentrations (100 – 1000 ppm), relates to data in Figure 4.

Polymer / shear stability

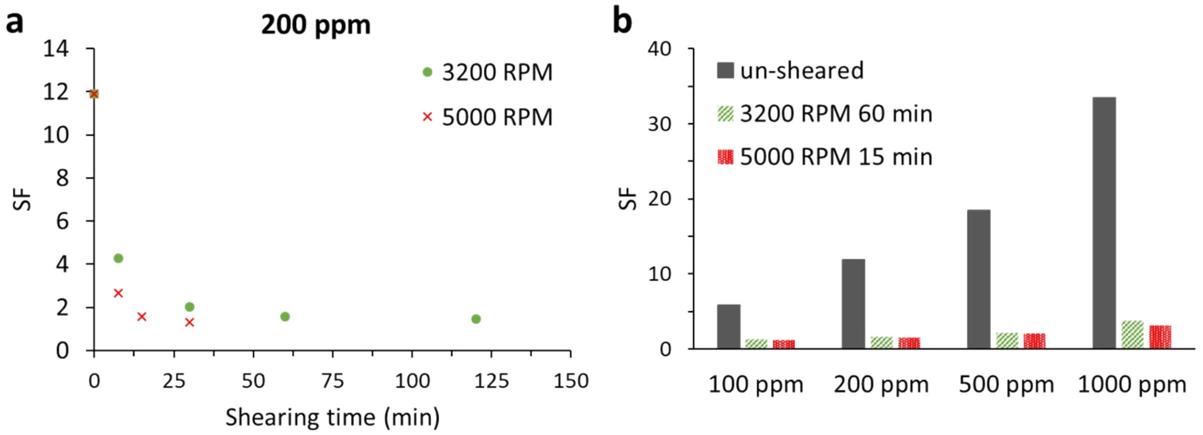

Figure S20. a) Effect of shear time (in Ultra-Turrax mixer, 100 mL sample volume) on Screen Factor for AM-5-6M at both 3200 and 5000 RPM. b) Screen Factor of AM-5-6M at 100 to 1000 ppm before and after standard shear treatments. Data relates to Figure 5.

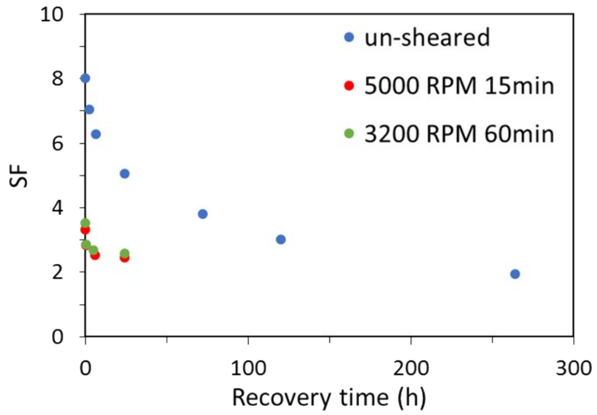

Figure S21. Shear treatment results for T1 Fe(II) at 1000 ppm showing no recovery in SF after shearing. Data relates to Figure 5.

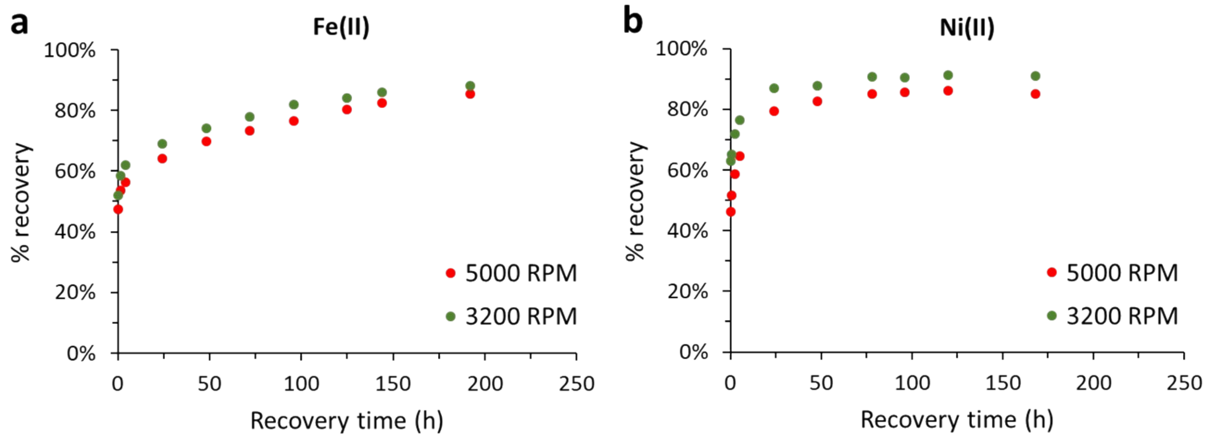

Figure S22. Shear recovery for a) Fe(II) and b) Ni(II) LCPs of T3 after shearing a 100 mL solution (1000 ppm) at either 5000 RPM for 15 minutes or 3200 RPM for 60 minutes (in the dark), expressed as a percentage of SF for an un-sheared sample after the same time. Data relates to Figure 5.

Experimental Procedures

Materials

Dry THF (99.9%, Merck) was obtained from a Solvent Purification System and stored under nitrogen over a sodium mirror prior to use. N,N-dimethylacrylamide (DMA, 99%) was obtained from Sigma Aldrich and passed through basic alumina prior to use to remove inhibitor.

Acetic acid (glacial, VWR Chemicals), acrylamide (AM, 98%, Aldrich), 2-acetylpyridine (99%, Aldrich), ammonium acetate (98%, Riedel-de Haën), carbon disulfide (CS_2, 99.9%, Aldrich), chloroform ($CHCl_3$, analysis grade, Merck), 4-chloroterpyridine (99%, Aldrich), diethyl ether (RCI Labscan), 1,8-diazabicyclo[5.4.0]undec-7-ene (DBU, 98%, Aldrich), diisopropyl azodicarboxylate (DIAD, 98% Aldrich), dimethyl sulfoxide (DMSO, 99.9%, Merck), ethanol (analysis, Merck), ethyl acetate (99.5%, Merck), 2-mercaptoethanol (98%, Aldrich), petroleum spirit (Merck), polyacrylamide (5000 – 6000 kDa, Arcos organics), potassium hydroxide (KOH, 85% Merck), thionyl chloride (97%, Aldrich), triphenylphosphine (Ph_3P, 99%, Aldrich), $CuCl_2{\cdot}2H_2O$ (99%, Alfa Aesar), $CoCl_2{\cdot}6H_2O$ (reagent grade, Aldrich), $FeCl_2{\cdot}4H_2O$ (99%, Aldrich), $NiCl_2{\cdot}4H_2O$ (98%, Merck) and $ZnCl_2$ (98%, Aldrich) were all used as received.

4-(hydroxymethyl)benzaldehyde[13] was synthesised according to literature procedures with analytical data in agreement with published values. Samples of paraffinic oil and guar drift control adjuvants were provided by Nufarm Ltd. The chain transfer agent S,S′-bis(α,α′-dimethyl-α″-acetic acid)trithiocarbonate (DMAT) was synthesised according to literature procedures,[2] with analytical data in agreement with published values.

RAFT agent synthesis

tpy-OH

Synthesis of 4′-(2-hydroxyethylsulfanyl)-2,2′:6′,2″-terpyridine (tpy-OH) was modified from a literature preparation for 4′-(2-hydroxyethoxy)-2,2′:6′,2″-terpyridine.[3] 2-Mercaptoethanol (2.0 mL, 28.5 mmol) was added to a stirred suspension of crushed KOH (1.44 g, 25.7 mmol) and dry DMSO (20 mL) under a nitrogen atmosphere. The suspension was then heated to 60°C for 15 minutes, which resulted in the KOH dissolving into the solution. 4-Chloroterpyridine (1.5 g, 5.6 mmol) was then added incrementally

over 1 h followed by a further 23 h of stirring at 60°C. An off-white solid was then precipitated by addition of chilled DI water (50 mL), which was filtered and washed with DI water (2 x 25 mL). After drying *in vacuo* (50°C), 1.65 g of off-white solid was isolated (95% yield), pure by ^{1}H NMR spectroscopy.

^{1}H NMR (400 MHz, $CDCl_3$): δ = 8.70 – 8.63 (m, 2H, Ar-H_a), 8.60 – 8.53 (m, 2H, Ar-H_b), 8.33 (s, 2H, Ar-H_c), 7.82 (dt, 2H, *J* = 5.1, 1.6 Hz, Ar-H_d), 7.31 (ddd, 2H, *J* = 7.3, 4.8, 0.9 Hz, Ar-H_e), 3.99 – 3.89 (br m, 2H, CH_2-H_f), 3.37 (t, 2H, J = 6.3, CH_2-H_g), 2.89 – 2.79 (br m, 1H, OH-H_h).

^{13}C NMR (100 MHz, $CDCl_3$): δ = 155.9, 155.2, 150.3, 149.2, 137.1, 124.1, 121.6, 118.2, 60.7, 33.9

HRMS (ESI) calc. for $C_{17}H_{16}N_3OS$ $[M+H^+]$ m/*z* = 310.1014, found m/*z* = 310.1013.

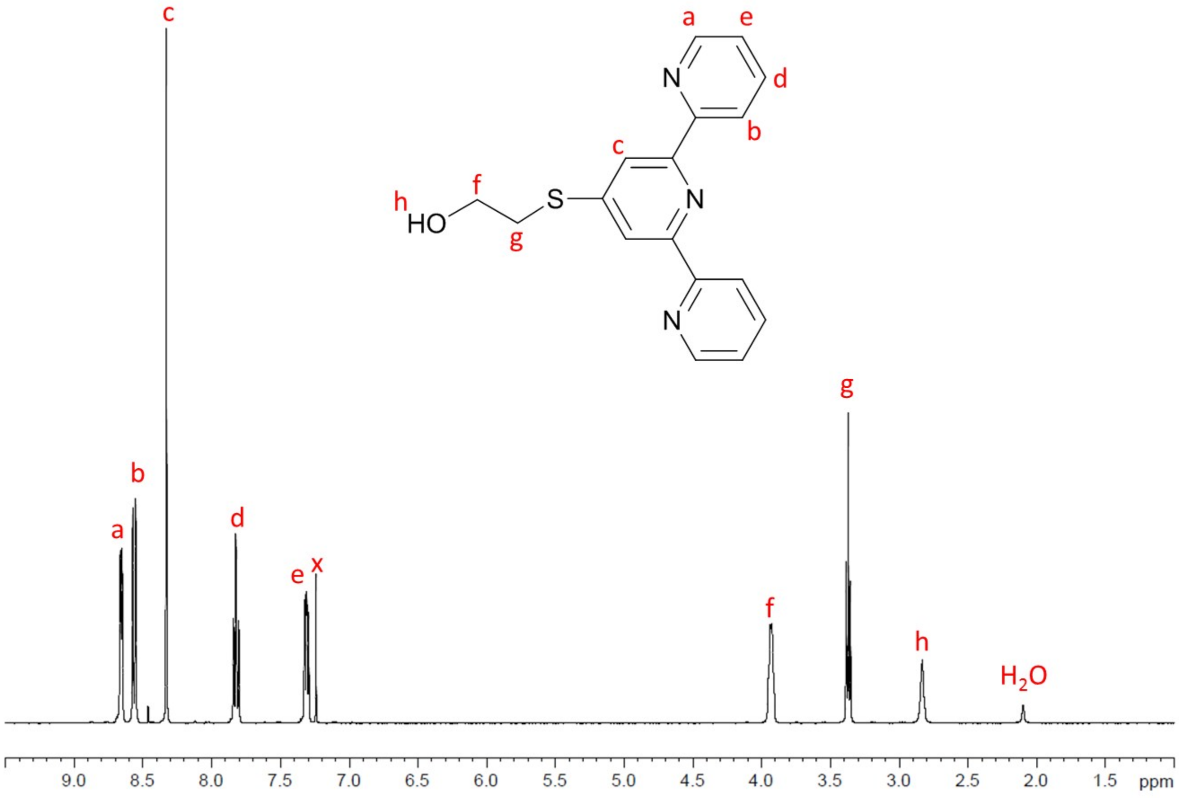

Figure S23. ^{1}H NMR (400 MHz, $CDCl_3$) spectrum of tpy-OH.

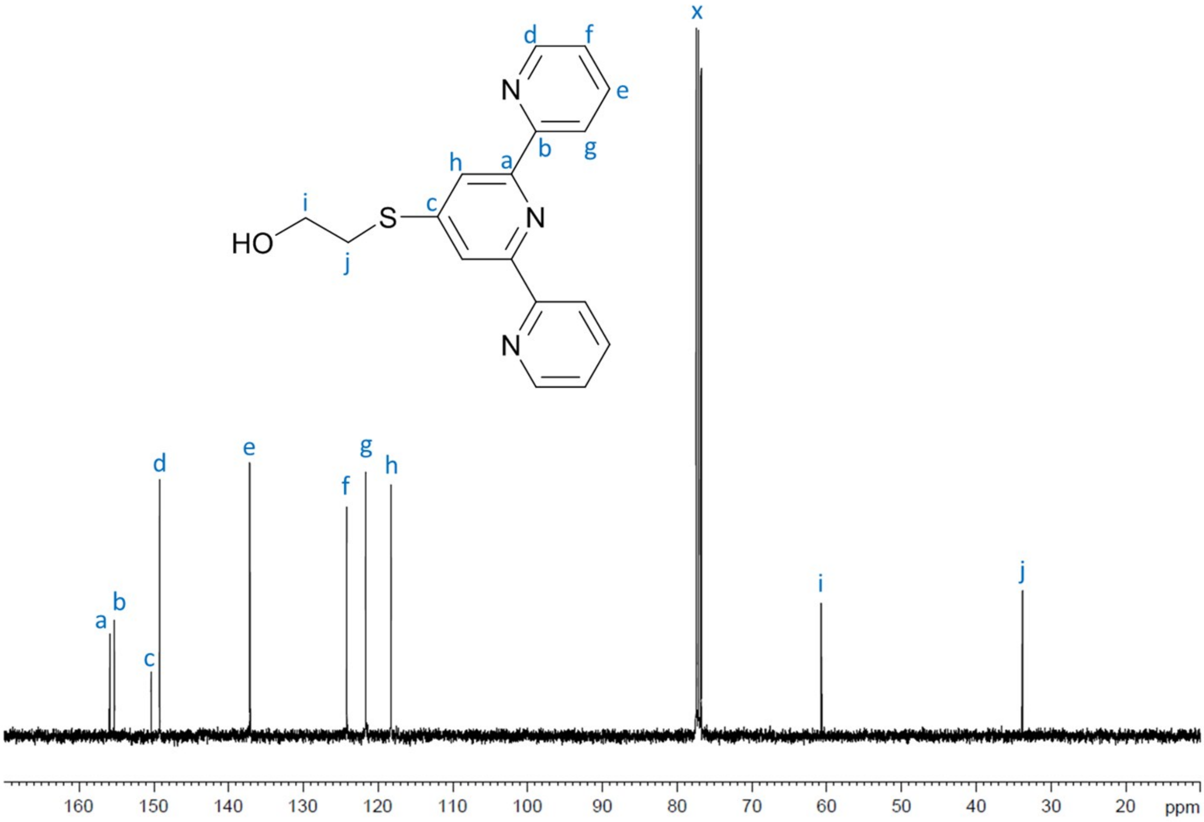

Figure S24. ^{13}C NMR (100 MHz, $CDCl_3$) spectrum of tpy-OH.

tpyDMAT

Synthesis of bis[(4′-(sulfanylethyl) 2,2′:6′,2″-terpyridine) 2,2-dimethylacetate] trithiocarbonate (tpyDMAT) was conducted using Mitsunobu coupling conditions adapted from literature.[4] DMAT (675 mg, 2.39 mmol), triphenylphosphine (Ph_3P, 2.29 g, 8.73 mmol) and a slight molar excess (relative to the carboxylic acid functionalities on DMAT) of tpy-OH (1.55 g, 5.02 mM) were combined; and the reaction flask was purged with nitrogen before adding dry THF (25 mL) via cannula. After mild heating to 40°C to dissolve all components, the bottom of the flask was placed into an ice bath (with stirring) and diisopropyl azodicarboxylate (DIAD, 1.3 mL, 6.7 mmol) was added over 30 minutes by syringe pump. Once the addition was complete, the reaction was removed from the ice bath and stirred for a further 22 h at room temperature. The crude product was purified by column chromatography (SiO_2: diethyl ether/petroleum spirit 1:2 up with polarity gradually increased to neat ethyl acetate). To completely remove the Ph_3PO by-product further purification by column chromatography was required, giving 1.56 g of a yellow glassy solid (75% yield) after drying *in vacuo*.

^{1}H NMR (400 MHz, $CDCl_3$): δ = 8.69 – 8.63 (m, 4H, Ar-H_a), 8.59 – 8.53 (m, 4H, Ar-H_b), 8.31 (s, 4H, Ar-H_c), 7.81 (dt, 4H, J = 5.1, 1.7 Hz, Ar-H_d), 7.29 (ddd, 4H, J = 7.4, 4.8, 1.0 Hz, Ar-H_e), 4.37 (t, 4H, J = 6.4 Hz, CH_2-H_f)), 3.37 (t, 4H, J = 6.4 Hz, CH_2-H_g), 1.59 (s, 12H, CH_3-H_h).

^{13}C NMR (100 MHz, $CDCl_3$): δ = 218.8, 172.7, 155.9, 155.3, 150.3, 149.2, 137.0, 124.1, 121.5, 118.1, 64.2, 56.1, 29.5, 25.2

HRMS (ESI) calc. for $C_{43}H_{40}N_6O_4S_5$ [M+H$^+$] m/z = 865.1787, found m/z = 865.1791

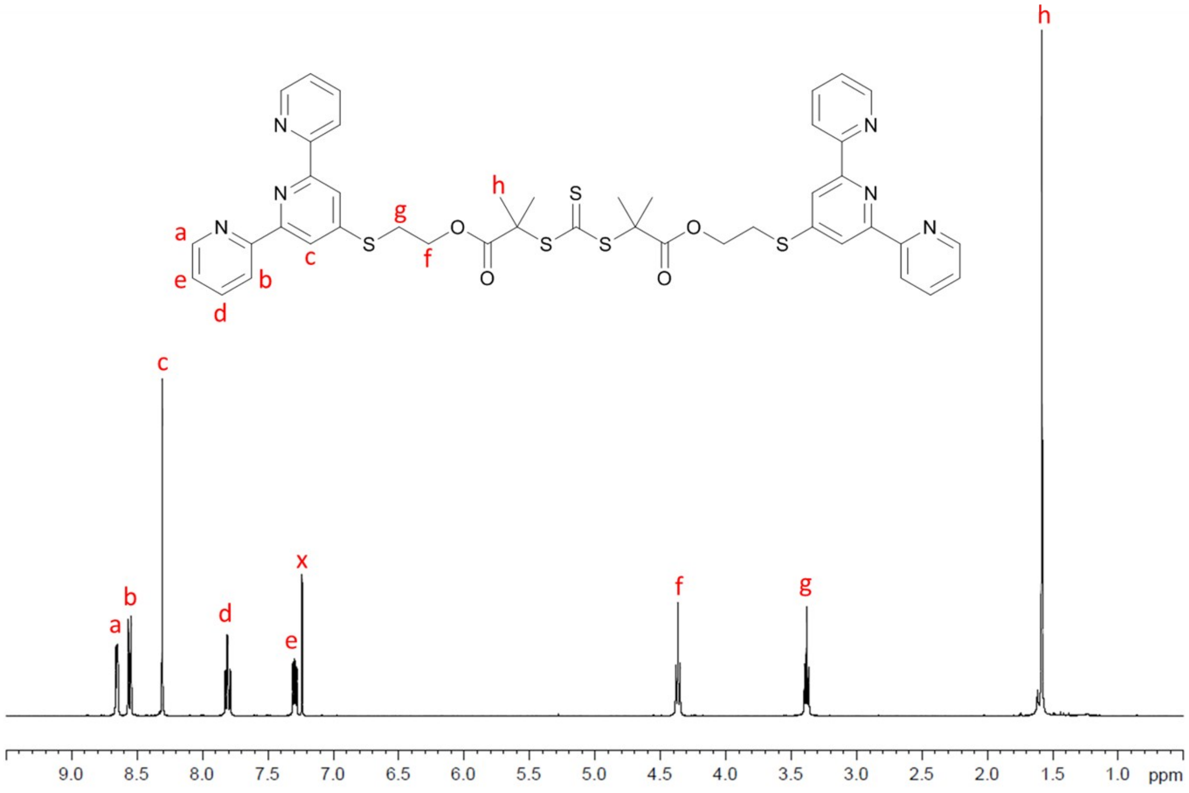

Figure S25. ^{1}H NMR (400 MHz, $CDCl_3$) spectrum of tpyDMAT.

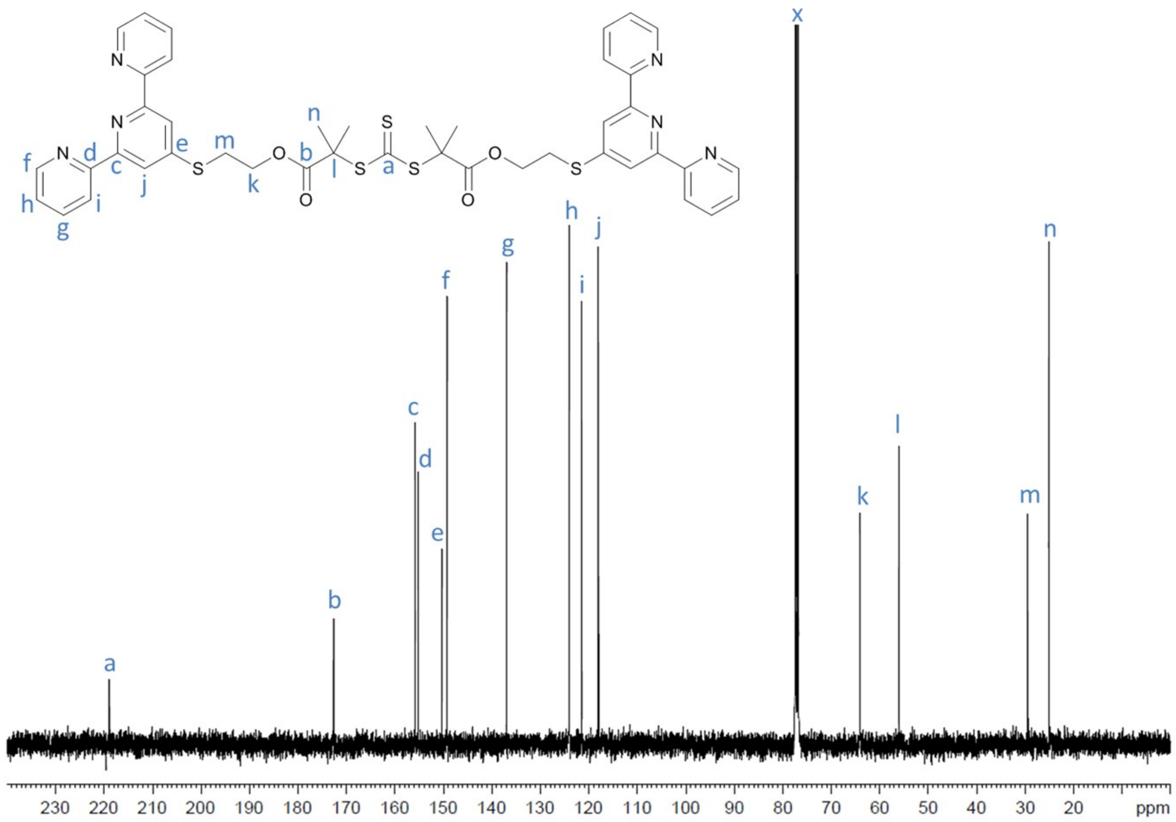

Figure S26. ^{13}C NMR (100 MHz, $CDCl_3$) spectrum of tpyDMAT.

tpyTTC synthesis overview

The synthesis for tpyTTC has already been reported,[5] however a modified procedure was implemented as will be described below (**Scheme S2**). A benzyl alcohol functionalised terpyridine (tpy-BnOH) was first synthesised in relatively modest yield (39%) by a solvent-free method which has been previously demonstrated for other benzyl functionalised terpyridines. This synthesis is most efficiently conducted in one step (Method A, **Scheme S2**), without isolating the intermediate ((Ac-Py)$_2$BnOH). The low yield for this reaction is most likely due to the formation of multiple by-products during the formation of (Ac-Py)$_2$BnOH. The benzyl alcohol (tpy-BnOH) was then converted to the benzyl chloride (tpy-BnCl), which was reacted with CS_3^{2-} to form the desired terpyridine-functionalised trithiocarbonate (tpyTTC). The dianion CS_3^{2-} was formed *in-situ* by reaction of CS_2 with DBU in DMSO, similar yields with longer reaction times were also obtained when KOH was used instead of DBU.

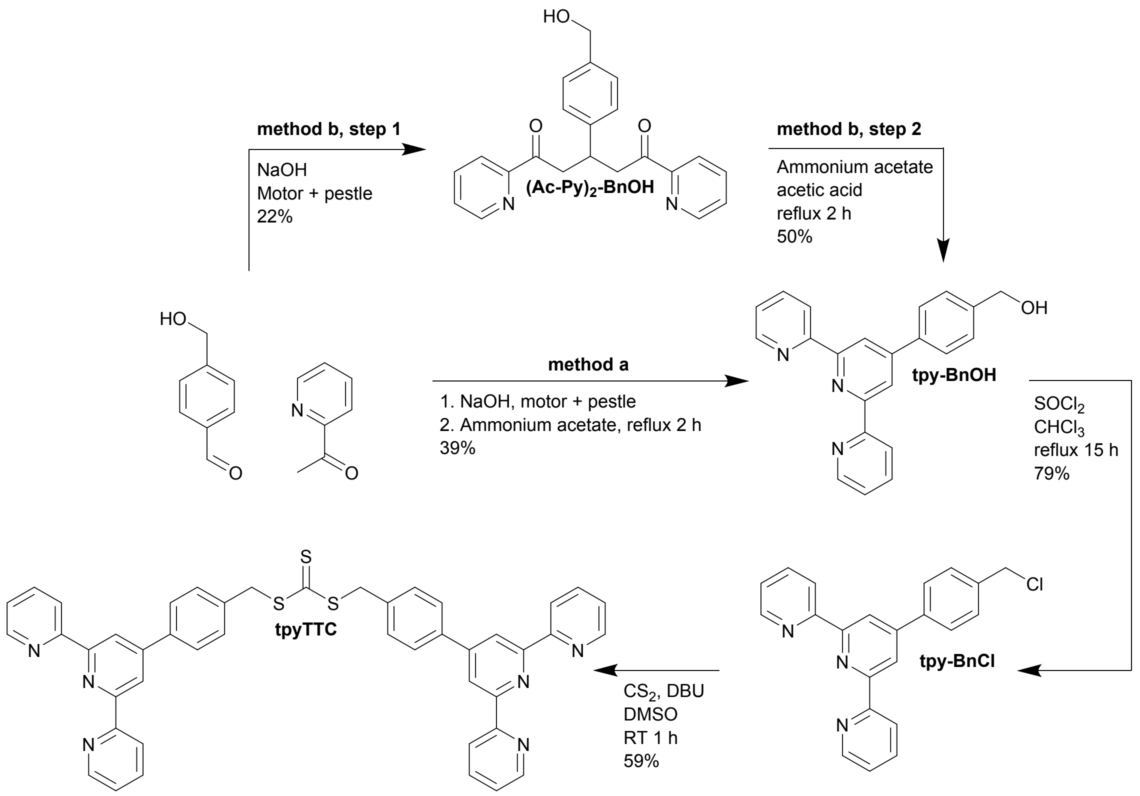

Scheme S2. Synthesis of tpyTTC RAFT agent.

(Ac-Py)$_2$-BnOH

Synthesis of 3-(4-(hydroxymethyl)phenyl)-1,5-di(pyridin-2-yl)pentane-1,5-dione ((Ac-Py)$_2$-BnOH) was conducted according to a modified procedure from literature.[6] A fine powder of NaOH (0.60 g, 15 mmol) was first made in a mortar and pestle. A solution of 4-(hydroxymethyl)benzaldehyde (1.00 g, 7.35 mmol) dissolved in 2-acetylpyridine (1.79 g, 14.8 mmol) was added to the powder, forming a sticky yellow paste which after grinding for approximately 10 minutes formed a fine yellow powder. After further aggregation (1 h), the powder was dissolved in ethyl acetate (80 mL) and washed with DI water (2 x 80 mL). The crude mixture was then purified by column chromatography (SiO_2: petroleum spirit/ethyl acetate 1:2), yielding 590 mg of a milky white crystalline solid after drying *in vacuo* (22% yield).

^{1}H NMR (400 MHz, $CDCl_3$): δ = 8.66 (dq, 2H, J = 4.8, 0.9 Hz, Ar-H$_a$), 7.97 (dt, 2H, J = 7.8, 1.0 Hz, Ar-H$_b$), 7.81 (td, 2H, J = 5.1, 1.8 Hz, Ar-H$_c$), 7.46 (ddd, 2H, J = 7.6, 4.8, 1.2 Hz, Ar-H$_d$), 7.43 – 7.37 (m, 2H, Ar-H$_e$), 7.29 – 7.25 (m, 2H, Ar-H$_f$), 4.63 (d, 2H, J = 5.7 Hz, CH$_2$-H$_g$), 4.17 (quintet, 1H, J = 7.2

Hz, CH-H_h), 3.79 (dd, 2H, J = 17.6, 7.6 Hz, CH_2-H_i), 3.65 (dd, 2H, J = 17.6, 6.7 Hz, CH_2-H_j), 1.54 (t, 1H, J = 6.0 Hz, CH_2-H_k).

^{13}C NMR (100 MHz, $CDCl_3$): δ = 200.1, 153.5, 149.0, 144.3, 139.0, 137.0, 128.1, 127.4, 127.2, 122.0, 65.4, 44.3, 36.0.

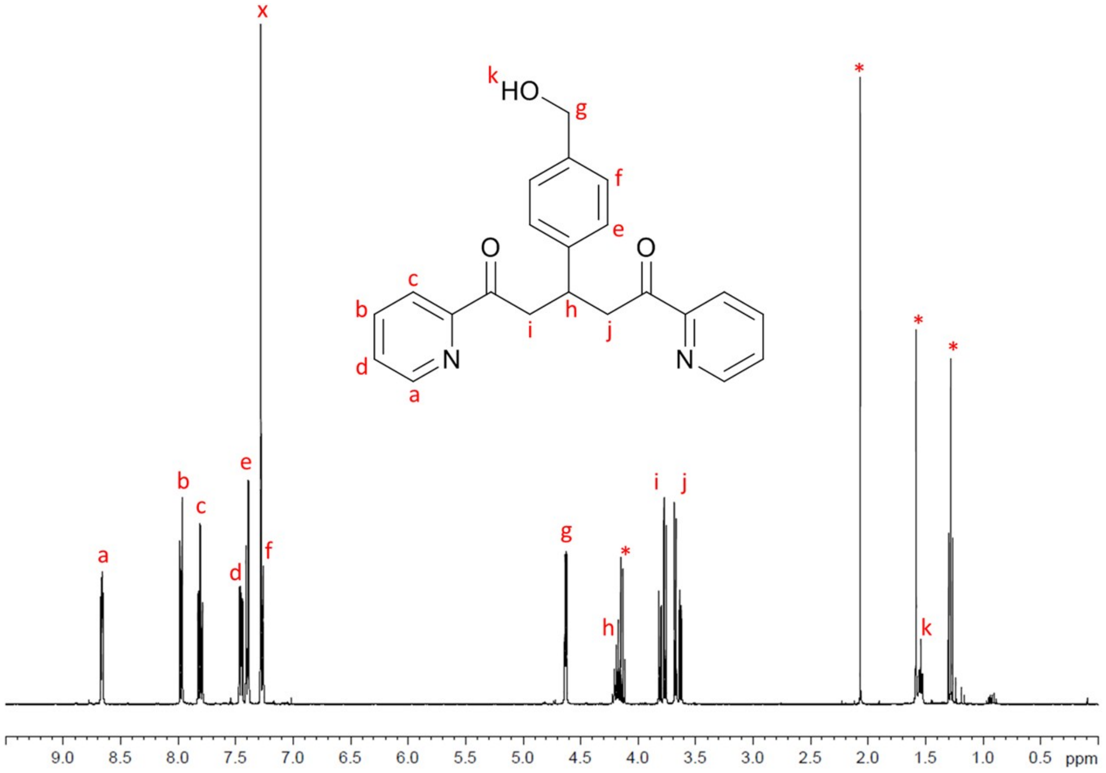

Figure S27. ^1H NMR (400 MHz, $CDCl_3$) spectrum of $(Ac\text{-}Py)_2$-BnOH. *Residual ethyl acetate and water signals.

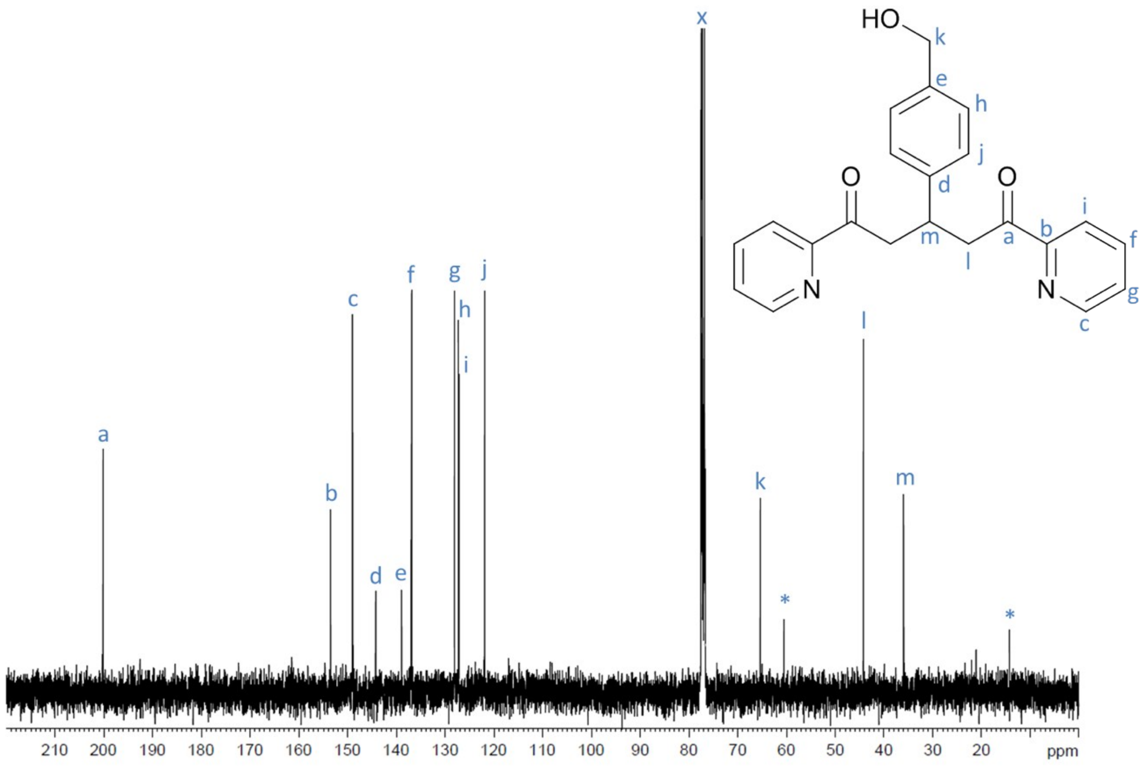

Figure S28. ^{13}C NMR (100 MHz, $CDCl_3$) spectrum of $(Ac\text{-}Py)_2$-BnOH. *Residual ethyl acetate signals.

tpy-BnOH

(Method A)

Synthesis of 4′-(4-hydroxymethylphenyl)-2,2′:6′2″-terpyridine (tpy-BnOH) was conducted according to a modified procedure from literature.[6] A fine powder of NaOH (1.2 g, 30 mmol) was first made in a mortar and pestle. A solution of 4-(hydroxymethyl)benzaldehyde (2.00 g, 14.7 mmol) dissolved in 2-acetylpyridine (3.56 g, 29.4 mmol) was added to the powder, forming a sticky yellow paste which after grinding for approximately 15 minutes formed a fine yellow powder. This was then combined with ammonium acetate (7.5 g, 97 mmol) and glacial acetic acid (30 mL), followed by heating to reflux (2 h). The crude mixture was then concentrated *in vacuo* before water (50 mL) was added to precipitate out a tarry solid. This crude solid contains the acetate ester of the product, which was converted back to the alcohol by dissolving in THF/ethanol (50/50) and adding 2.0 M NaOH (10 mL). Volatile solvents were then removed *in vacuo*, followed by further additions of water, forming a filterable brown solid. This was then washed with ethanol and water, giving 1.93 g solid after drying in a vacuum oven (39% yield). ^{1}H NMR spectroscopic analysis was in agreement with literature values.[17]

(Method B)

In an alternative method (for the synthesis of tpy-BnOH),[6] $(Ac-Py)_2$-BnOH (590 mg, 1.64 mmol), ammonium acetate (520 mg, 6.75 mmol) and glacial acetic acid (3.0 mL) were refluxed (2 h) forming a dark burgundy coloured solution. 1.0 M NaOH (70 mL) was then added, forming of a mix of yellow and white solids. These solids were collected, re-dissolved in THF/ethanol (50/50, 40 mL) and 1.0 M NaOH (15 mL). The volatile solvents were removed *in vacuo*, with the solids washed further with DI water/ethanol (80/20). A salmon orange powder was then obtained after drying in a vacuum oven (275 mg, 50% yield).

^{1}H NMR (400 MHz, DMSO-d_6): δ = 8.77 – 8.71 (m, 2H, Ar-H_a), 8.69 (s, 2H, Ar-H_b), 8.64 (d, 2H, *J* = 8.0 Hz, Ar-H_c), 8.00 (t, 2H, *J* = 7.4 Hz, Ar-H_d), 7.87 (d, 2H, *J* = 8.0 Hz, Ar-H_e), 7.54 – 7.46 (m, 4H, Ar-H_{f+g}), 5.31 (t, 1H, *J* = 5.6 Hz, OH-H_h), 4.57 (d, 2H, *J* = 5.4 Hz, CH_2-H_i).

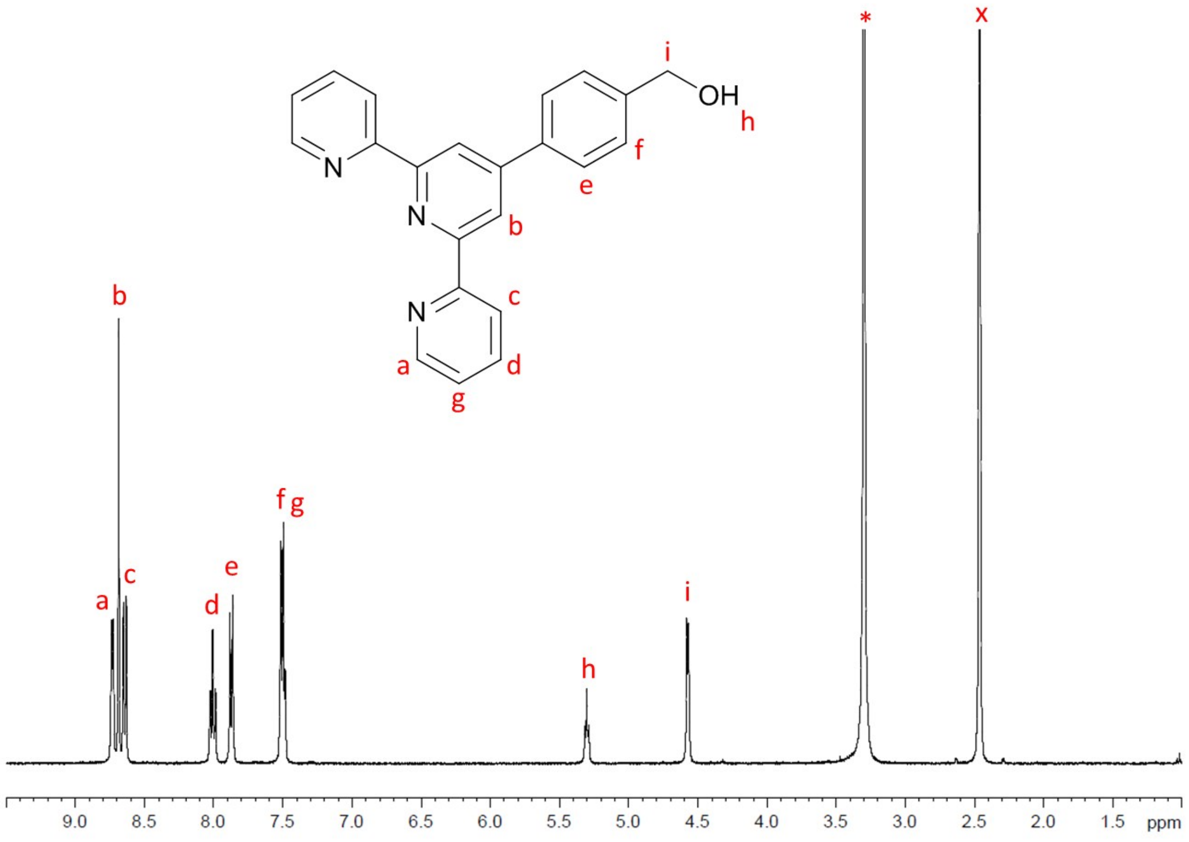

Figure S29. ^{1}H NMR (400 MHz, DMSO-d_6) spectrum of tpy-BnOH. *Residual water signal.

tpy-BnCl

Synthesis of 4′-(4-chloromethylphenyl)-2,2′:6′2″-terpyridine (tpy-BnCl) was modified from an existing literature procedure.[7] Thionyl chloride (1.4 mL, 19.3 mmoL) was added to a suspension of tpy-BnOH

(1.93 g, 5.69 mmol) in chloroform (50 mL) and heated to mild reflux under nitrogen atmosphere. After 20 h the solution was washed with aqueous sodium bicarbonate solution (4 wt%, 50 mL) and brine (100 mL). The organic layer was then dried over Na_2SO_4 and the volatiles removed *in vacuo* yielding 1.60 g of a faint brown solid (79% yield).

^{1}H NMR (400 MHz, $CDCl_3$): δ = 8.75 – 8.72 (m, 4H, Ar-H_{a+b}), 8.70 – 8.65 (m, 2H, Ar-H_c), 7.93 – 7.85 (m, 4H, Ar-H_{d+e}), 7.53 (d, 2H, *J* = 8.3 Hz, Ar-H_f), 7.36 (ddd, 2H, *J* = 7.4, 4.8, 1.2 Hz, Ar-H_g) 4.67 (s, 2H, CH_2)

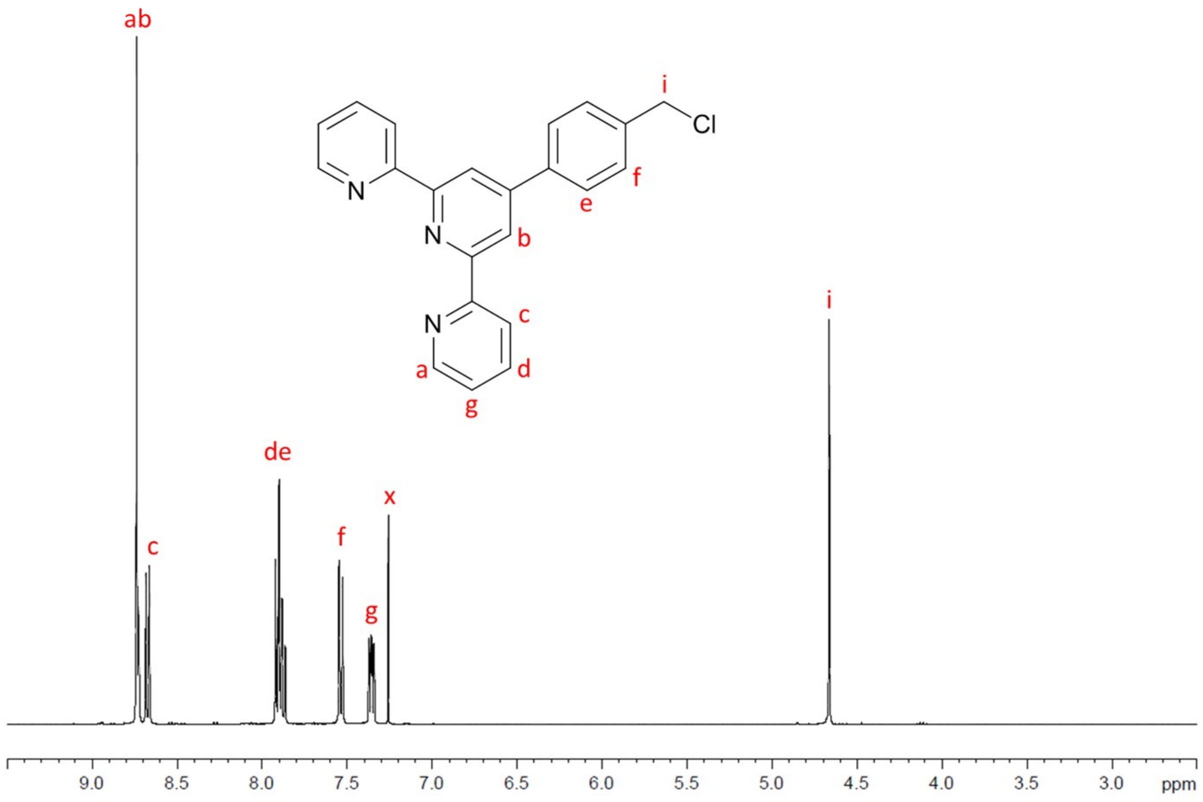

Figure S30. ^{1}H NMR (400 MHz, $CDCl_3$) spectrum of tpy-BnCl.

tpyTTC

Synthesis of bis(4′-(*p*-methylenephenyl)-2,2′:6′,2″-terpyridine) trithiocarbonate (tpyTTC) was modified from an existing literature procedure.[5] CS_2 (412 mg, 5.41 mmol), DBU (682 mg, 4.47 mmol), DMSO (3 mL) and DI water (35 μL) were combined and stirred for 30 minutes forming a deep red

coloured solution (trithiocarbonate di-anion). Tpy-BnCl (1.55 g, 4.34 mmol) was then added to the solution, with stirring continued for another 2 h. A crude brown solid was precipitated after addition of DI water (40 mL) which was then filtered and washed further with DI water (100 mL). This crude earthy solid was initially purified by re-crystallising from chloroform and was further treated by elution through a plug of neutral alumina (ethyl acetate / petroleum spirit 2:1). The solvent was removed *in vacuo*, yielding 955 mg of a yellow solid (59% yield).

^{1}H NMR (400 MHz, $CDCl_3$): δ = 8.75 – 8.71 (m, 8H, Ar-H_{a+b}), 8.67 (dt, 4H, *J* = 7.9, 1.0 Hz, Ar-H_c), 7.91 – 7.84 (m, 8H, Ar-H_{d+e}), 7.50 (d, 4H, *J* = 8.3 Hz, Ar-H_f), 7.35 (ddd, 4H, *J* = 7.5, 4.8, 1.2 Hz, Ar-H_g), 4.72 (s, 4H, CH_2-H_h)

^{13}C NMR (100 MHz, $CDCl_3$): δ = 222.4, 156.3, 156.1, 149.8, 149.3, 138.2, 137.0, 136.1, 130.0, 127.8, 124.0, 121.5, 118.9, 41.4

MALDI-TOF MS calc. for $C_{45}H_{32}N_6S_3$ [M+H$^+$] m/*z* = 753.19, found m/*z* = 753.20

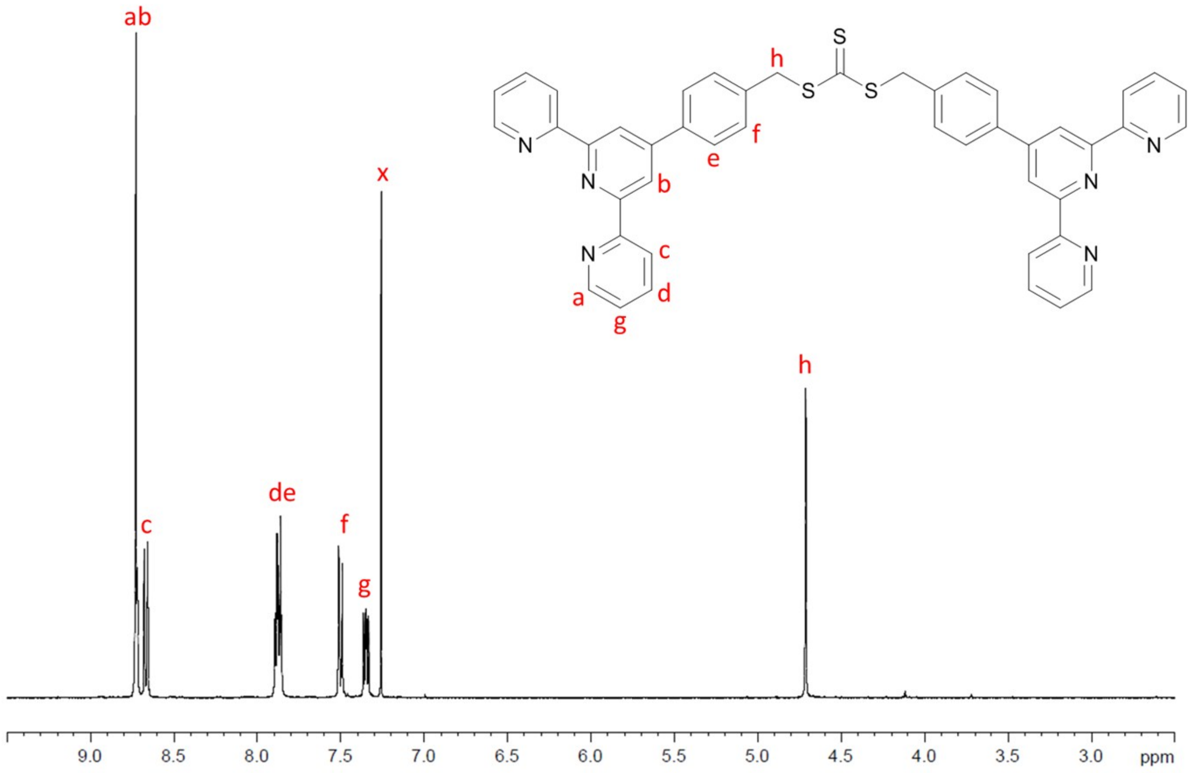

Figure S31. ^{1}H NMR (400 MHz, $CDCl_3$) spectrum of tpyTTC.

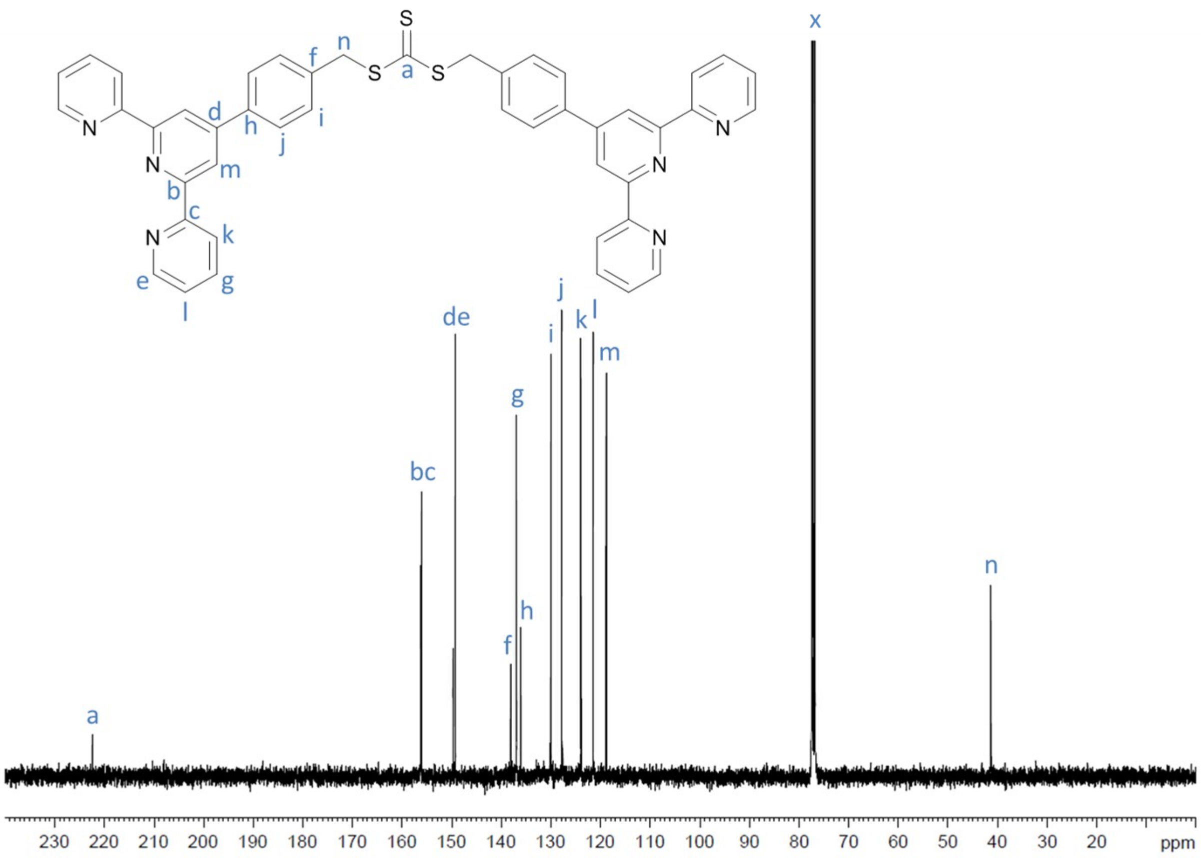

Figure S32. ^{13}C NMR (100 MHz, $CDCl_3$) spectrum of tpyTTC. Assignments from literature.[5]

Polymer synthesis

Typical macro-CTA synthesis procedure

The general procedure for macro-CTA synthesis is demonstrated for the synthesis of M1. TpyDMAT (575 mg, 0.665 mmol), AM (795 mg, 11.2 mmol) and DMSO (5.0 mL) were combined in a glass tube sealed with rubber septum. The reaction mixture was deoxygenated by bubbling with argon for 15 minutes and placed into the LED reactor (402 nm, 52 W). The reaction was quenched after 90 minutes irradiation (79% conversion by ^{1}H NMR spectroscopy) by removing the glass tube from the light source and opening to air. The polymer was then purified by precipitation into acetone. $M_{n,NMR}$ = 1.82 kDa.

A similar procedure was followed for M2 and M3, with dialysis (Spectra/Por® 6 Standard RC tubing, MWCO 1 kDa) used to remove monomer instead of precipitation.

Typical polymer synthesis procedure

The general procedure for (chain-extended) polymer synthesis is demonstrated for the synthesis of T1. M1 (12.9 mg, 0.0071 mmol), AM (3.55 g, 49.9 mmol), DI water (18.75 mL), dioxane (1.25 mL) and 1.0 mM TFA (5.0 mL) were combined in a glass tube sealed with rubber septum. The small addition of acid was implemented to reduce the pH to ~ 3.7, as initial attempts at approximately neutral pH indicated that ester hydrolysis (on tpyDMAT polymers) was leading to poor retention of end-groups. The reaction mixture was deoxygenated by bubbling with nitrogen for 30 minutes and placed into the LED reactor (451 nm, 24 W). The reaction was quenched after 26 h irradiation (81% conversion by ^{1}H NMR spectroscopy) by removing the glass tube from the light source and opening to air. The polymer was then purified by dialysis using Spectra/Por® 6 Standard RC tubing (MWCO 25 kDa). $M_{n,conv}$ = 401 kDa.

Polymerisation conversion (ρ) was calculated by comparing the ^{1}H NMR integrals of the monomer unsaturated protons (∫M: 5.5 – 6.3 ppm for AM, 5.5 – 6.7 ppm for DMA) to the polymer backbone (∫P: 1.1 – 2.5 ppm for PAM, 1.1 – 3.2 ppm for PDMA) (**Equation S1**).

$$\rho = \frac{\int P}{\text{[illegible]}}$$ **Eq. S1**

$M_{n,conv}$ was calculated according to **Equation S2** where $[M]_0$ is the initial monomer concentration, $[CTA]_0$ is the initial chain transfer agent (CTA) concentration and M_M and M_{CTA} are the monomer and CTA molecular weights, respectively.

$$M_{n,conv} = \rho \times \frac{[M]_0}{[CTA]_0} \times M_M + M_{CTA} \qquad \textbf{Eq. S2}$$

$M_{n,NMR}$ was calculated by integrating the ^{1}H NMR RAFT end-group signals in a manner dependent on the RAFT agent used. For example, AM polymers of DMAT have $C(CH_3)_2$ signals at 0.94 – 1.05 ppm and when integrated to 12, they represent the theoretical structure of one RAFT controlled polymer chain. Similarly, the end-groups on polymers made from other RAFT agents synthesised during this work were integrated to represent one RAFT controlled polymer chain (terpyridine aromatic protons approx. 7.0 – 9.0 ppm). The polymer backbone signals (CH and CH_2 groups for PAM 1.1 – 2.5 ppm, CH, CH_2 and CH_3 groups for PDMA 1.1 – 3.2 ppm) were then integrated to determine DP_{NMR}, which was used in **Equation S3** to evaluate $M_{n,NMR}$.

$$M_{n,NMR} = DP_{NMR} \times M_M + M_{CTA} \qquad \textbf{Eq. S3}$$

$M_{n,UV}$ was calculated by measuring the number of terpyridine end-groups by UV-Vis spectroscopy on the Fe(II) complex (using the appropriate extinction coefficient measured in **Figures S8 – S10**). This was then related to $M_{n,UV}$ by assuming an end-group fidelity of 100% (two terpyridines per polymer chain, **Equation S4**). A significantly inflated $M_{n,UV}$ results when a lower than expected amount of terpyridine end-groups are present, indicative of poor end-group fidelity.

$$M_{n,UV} = \frac{2 \times \varepsilon_{566nm}(M^{-1}cm^{-1}) \times C_{polymer}(gL^{-1}) \times L(cm)}{A_{566nm}} \qquad \textbf{Eq. S4}$$

Where $C_{polymer}$ = concentration of polymer and L = path length during Abs measurement. Note for tpyDMAT polymers ε_{566} = 9536 $cm^{-1}M^{-1}$ and for tpyTTC derived polymers ε_{566} is replaced by ε_{568nm} = 11 740 $cm^{-1}M^{-1}$. The error value reported is the SD across absorbance data for that polymer.

Coordination polymer synthesis

LCPs were formed at ambient conditions by adding precise amounts of transition metal ion stock solutions to a solution of the un-complexed polymer at known concentration (typically 10 or 1 mg/mL). The amount of a transition metal ion needed to maximise bis-complexation and LCP molecular weight was determined by UV-Vis spectroscopy monitored titrations. Since the concentration at which the LCP was formed can have a significant effect on the resultant molecular weight,[8] the specific concentration used for each synthesis will be specified in the results. After transition metal ion addition, the solutions were mixed on an orbital shaker (IKA KS 501 Digital Orbital Shaker) for 1 hour before conducting analysis on the LCP, as the complex was found to be fully formed after this time (**Figure S33**).

All transition metal ion stock solutions (typically 4 mM) were prepared from the chloride salts of each metal ion ($FeCl_2$, $CuCl_2$, $ZnCl_2$, $CoCl_2$, $NiCl_2$) of varying levels of hydration. In the case of Fe(II), the stock solutions were prepared with 0.01 M HCl (aq.) instead of DI water under an argon atmosphere to limit oxidation to Fe(III) (oxidation less favoured for aqueous Fe(II) under acidic conditions).[9]

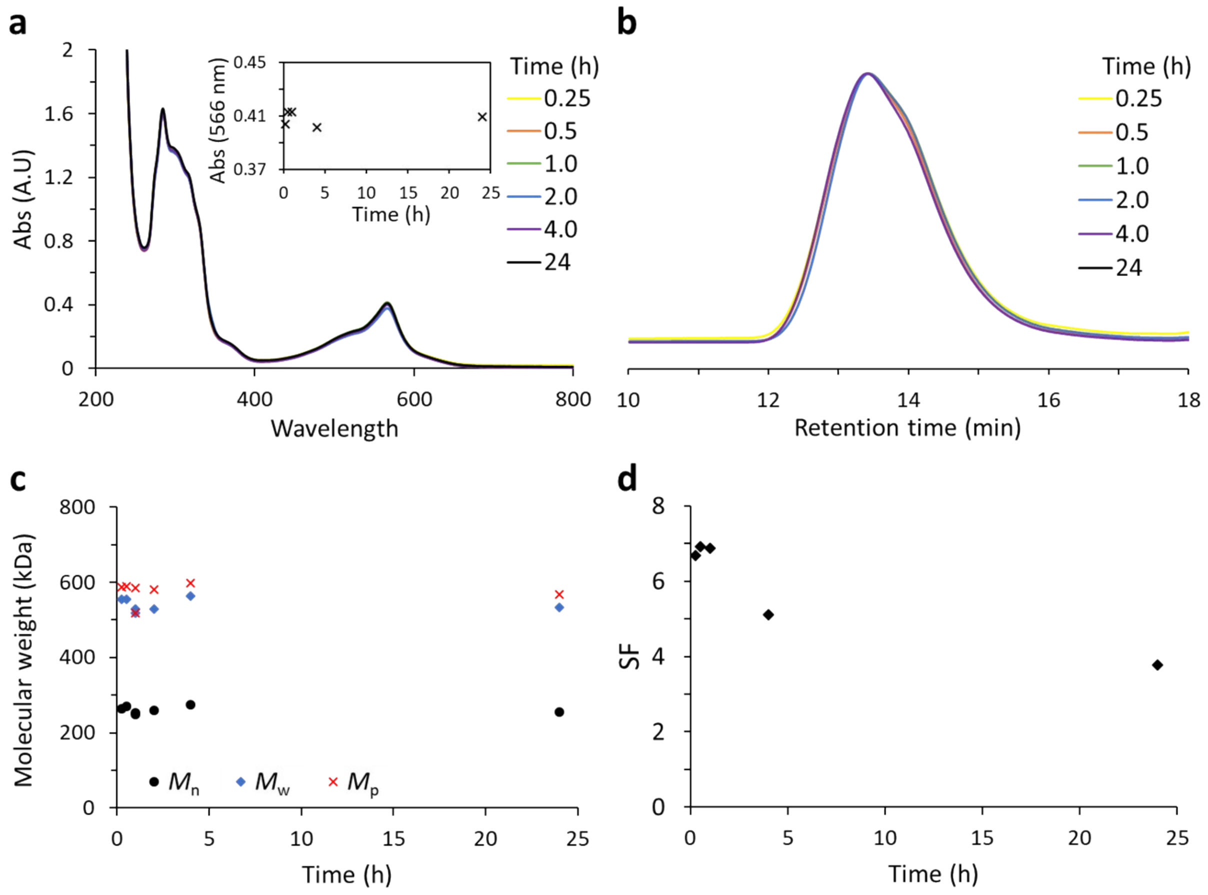

Figure S33. Effect of Fe(II) mixing time on LCP formation and molecular weight for T2. LCP formation was characterised by a) UV-Vis spectroscopy (10 mg/mL), GPC (1 mg/mL, b) traces and c) molecular weight data), and d) Screen Factor (1 mg/mL). Little difference was observed across all mixing times indicating that 1 hour is sufficient formation time.

NMR spectroscopy

^{1}H NMR and ^{13}C NMR spectra were recorded on Bruker AV-400 and AV-500 spectrometers (400 and 500 MHz respectively).

UV-Vis spectroscopy

UV-Vis absorbance spectra were obtained using an Agilent Technologies Cary 60 UV-Vis spectrophotometer. For each measurement a 1 cm path length quartz cuvette was used at a medium scan speed over 200 – 800 nm.

Visible light sources

Three different photo-reactors were constructed in our laboratory and their respective emission spectra were measured using a Solar Simulator Spectroradiometer from PV Measurements with wavelength range from 350 to 1100 nm (**Figure S34**). The first reactor (LED reactor 1) was constructed using ACULED® VHL™ (Very High Lumen) LEDs procured from Excelitas Technologies with λ_{max} measured as 402 nm. These LEDs were housed in a rectangular aluminium box with 4 banks of 9 LEDs surrounding a central cavity in which a small glass tube with the desired reaction mixture can be loaded. The second reactor (LED reactor 2) was constructed from a 4 meter strip of 246 SMD 5050 LEDs procured from eBay with λ_{max} measured to be 451 nm. These LEDs were wound around the inside of a plastic jar of diameter 9 cm. The third reactor (LED reactor 3) was constructed from a 5 meter strip of 300 RGB SMD 5050 LEDs procured from eBay with λ_{max} of the blue lights measured to be 444 nm. These LEDs were wound around the inside of a plastic jar of diameter 9 cm.

The average emission intensity of each photo-reactor was also measured to relate the input electrical power to the resultant emission intensity (**Figure S35**). For LED reactors 2 and 3 (λ_{max} = 451 and 444 nm, respectively), the power output was measured in the middle of the reactor using a Powermax 5200 laser power meter. For LED reactor 1 (λ_{max} = 402 nm), where the small central cavity limits the measurement position, a Silverline all UV radiometer (230 – 410 nm) was used with the diode held in the middle of the reactor. Throughout this work the LED power is specified as the electrical power supplied to the reactor.

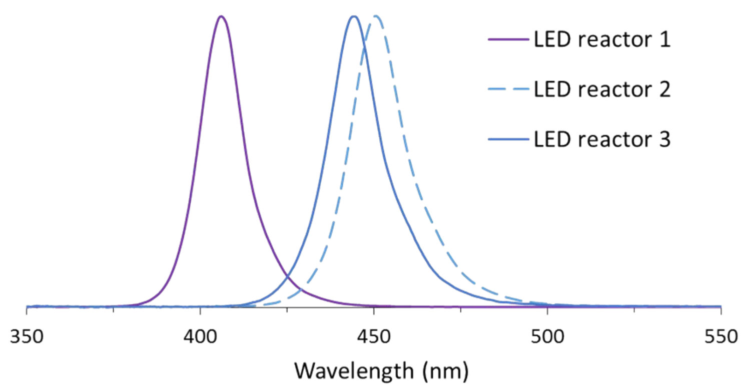

Figure S34. Relative emission spectra for LED reactors used in polymer synthesis.

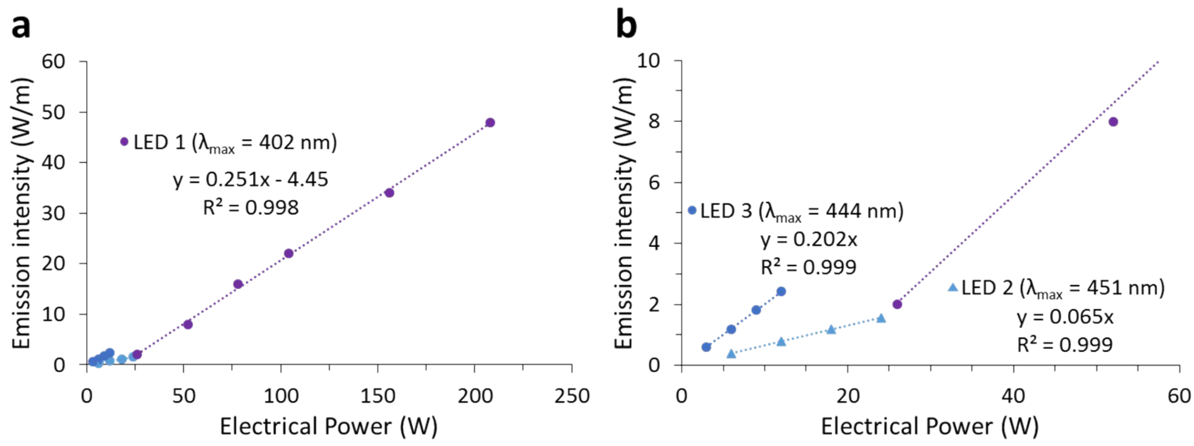

Figure S35. Emission intensity data for LED reactors used in this work as a function of electrical power supplied to the LEDs. a) Zoomed out plot highlighting data the for LED reactor 1, b) zoomed in plot highlighting data for LED reactors 2 and 3.

Gel permeation chromatography

The average molecular weight and dispersity (*Đ*) of the resultant polymers was measured through GPC. Most commonly (T2 – T4 and their Fe(II) complexes, C1 – C4), samples were analysed on a Waters Alliance aqueous system equipped with an Alliance 2695 Separations Module (integrated quaternary solvent delivery, solvent degasser and autosampler system), a Waters column heater module, a Waters 2414 RDI refractive index detector, a Waters PDA 2998 photodiode array detector (210 to 400 nm at 1.2 nm) and 2× Agilent PL-AquaGel-OH columns (Mixed H, 8 μm), each 300 mm × 7.8 mm^2, providing an effective molar mass range of 100 to 10^7 g/mol. Aqueous buffer was prepared containing 0.20 M $NaNO_3$, 0.01 M Na_3PO_4 in Milli-Q water with 200 ppm NaN_3 and adjusted to pH 8 and filtered through 0.45 μm filter. The filtered aqueous buffer was used as an eluent with a flow rate of 1.0 mL/min at 30°C. With the exception of work conducted in Section 4.3.1 (Chapter 4), samples were prepared in an identical buffer without sodium azide so as to minimise thiocarbonylthio RAFT group cleavage by sodium azide during sample preparation.[10] The GPC columns were calibrated with low dispersity PEO standards (Polymer Laboratories) ranging from 238 to 969 000 g/mol, and molar masses are reported as PEO equivalents. A 3rd-order polynomial was used to fit the log M_p vs. time calibration curve for both systems, which was near linear across the molar mass ranges.

Due to issues identified with column interactions of the terpyridine end-functionalised polymers (particularly at lower molecular weights), the polymerisation control study for synthesising T1 was monitored a Tosoh High Performance EcoSEC HLC-8320GPC System, which comprised of an autosampler, a vacuum degasser, a dual flow pumping unit, a Bryce-type refractive index (RI) detector, a UV detector set at 280 nm, a TSKgel SuperH-RC reference column, and three TSKgel PWXL columns (TSKgel G5000PWxL, TSKgel G6000PWxL and TSKgel MPWxL) connected in series. The analytical columns were calibrated with a series of poly(acrylic acid) (PAA) standards ranging from 1.06×10^3 to 1.52×10^6 g/mol. The eluent used was deionised water with 0.1 M $NaNO_3$ and 0.1 M $NaHCO_3$ (pH ≈ 8.3) at 40°C and at a flow rate of 1.0 mL/min.

Selected polymer samples were also analysed for absolute molecular weights on a Shimadzu-Wyatt system equipped with a CMB-20A controller system, a SIL-20A HT autosampler, a LC-20AD pump system, a DGU-20A degasser unit, a CTO-20A column oven, Shimadzu SPD-20A UV detector, a Wyatt Optilab rEX refractive index detector (RID), Wyatt Dawn Heleos II - 18 angle Multi Angle Light

Scattering (MALS) detector and 2× Waters Ultrahydrogel columns (250 and 2000, each 300 mm × 7.8 mm^2, providing an effective molar mass range of 10^3 to 10^6). Filtered aqueous buffer with 0.1 M $NaNO_3$ was used as an eluent with a flow rate of 0.8 mL/min at 40°C. Number (M_n) and weight average (M_w) molar masses were evaluated using Wyatt-Astra software. For each polymer type, the refractive index increment (dn/dc) was determined using at least five different polymer concentrations (1.0 to 10 mg/L) and the Wyatt OptiLab rEX RID in an offline mode. The dn/dc values were then entered for each polymer type, with absolute molecular weight and dispersity calculated from the Wyatt Astra software.

Viscosity measurements

Polymer solution viscosity was measured using a Discovery HR-3 hybrid rheometer with Peltier Plate accessory and a 60 mm 0.5° cone plate geometry attached. Each measurement was collected at 20°C and began with a 60 seconds pre-shear at 10 s^{-1}, followed by a flow sweep from 1 – 1000 s^{-1} over 5 minutes. The relative viscosity was then determined by dividing the viscosity of the polymer solution (averaged value over data points from the flow sweep that did not have a shear rate dependence) by the viscosity measured for DI water. Two independent samples from each polymer were analysed, with the standard deviation evaluated between the determined viscosities of each repeat.

The intrinsic viscosity of selected polymer samples was measured using a Discovery HR2 hybrid rheometer with 60 mm parallel plate geometry and solvent trap attached. A shear rate of 1 – 1000 s^{-1} was applied across all samples. The zero shear viscosity was then evaluated for each sample (η_0, viscosity for which there is no shear rate dependence) and pure water ($\eta_{solvent}$) allowing for the calculation of reduced viscosity (**Equation S5**, where c is concentration of polymer (mg/mL)). The intrinsic viscosity is then defined as the reduced viscosity of a polymer extrapolated to a zero concentration.

$$\eta_r = \frac{\eta_0 - \eta_{solvent}}{c \times \eta_{solvent}} \qquad \textbf{Eq. S5}$$

Screen Factor water measurements

The experimental apparatus used to measure Screen Factor (SF) was constructed following ASTM specifications.[11] Specifically, the apparatus used in this study consisted of a stack of five 100 mesh stainless steel screens packed into a cylinder. This screen pack was then connected to a 25 mL pipette using silicon tubing. The test fluid was drawn through the screen pack into the pipette. Once filled, the fluid was then allowed to flow back through the screen pack and the elution time was recorded (t_p). This time was then divided by the time it takes for the same volume of DI water to pass (t_w) to determine SF (**Equation S6**). Each sample (typically ≥ 30 mL) was filtered before analysis, measured 3 times and then the apparatus was washed with fast running DI water. At the end of each day the apparatus was also washed with ethanol to minimise any biological growth on the screen pack. Using this protocol a consistent measurement for t_w across extended periods of time was obtained (**Figure S36**).

$$SF = \frac{t_p}{t_w} \qquad \textbf{Eq. S6}$$

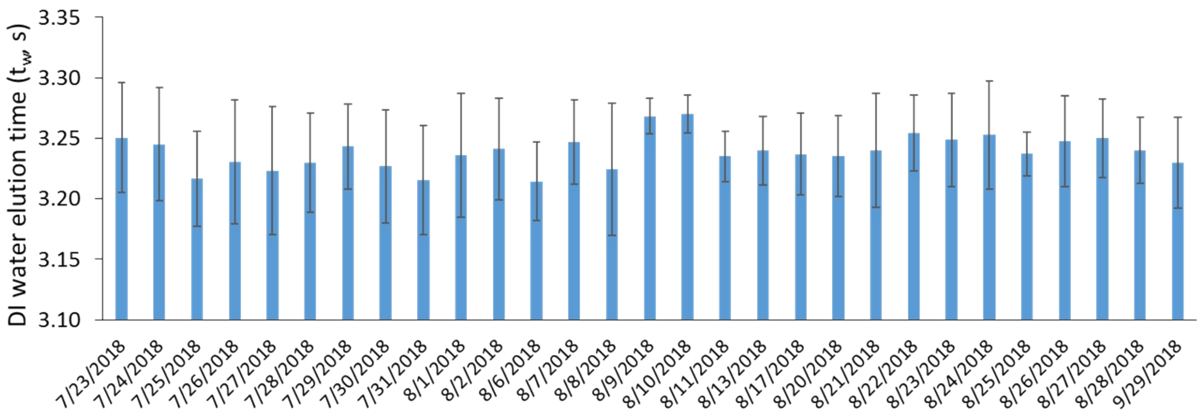

Figure S36. Variance in the elution time of DI water (t_w) through the screen pack for SF analysis over time (in total this data set represents 288 separate measurements).

Droplet size measurements

Droplet size analysis was conducted using a customised laboratory set-up, with a VisiSize P15 spray characterisation system located in an enclosed spray chamber. Each measurement was conducted by spraying 200 mL of solution through a XR 110 01 Flat Fan nozzle at 3.0 bar. The nozzle was connected to a high-pressure vessel containing the solution, pressurised by compressed air. After allowing 10 seconds for equilibration of pressure from the start of spraying, droplet size analysis was conducted over 10 seconds, collecting 147 frames of data. This analysis protocol was chosen to minimise the volume of solution needed per test, while maintaining good reproducibility between water-only tests. The system was flushed with water between analyses of different sample types (no flushing between the same adjuvant run at higher concentrations) until a droplet size distribution matching that expected for water only was recorded. The collected data was analysed on VisiSizer software, with between 200 and 5000 in-focus particles measured for each sample. The data has been presented in terms of V_{105}, with an artificially imposed air flow of 6.7 m/s. The V_{105} corresponds to the volume percent of the spray which has a droplet diameter less than 105 µm, which is often ascribed as being the fraction of the spray more likely to undergo spray drift, sometimes termed the driftable volume percentage. V_{150} is also often used for this measure, however since the nozzle used for this test produces a particularly fine droplet size distribution (selected to minimise sample volume), the finer definition was selected.

Supporting References

1. A. Rudin, in *The Elements of Polymer Science and Engineering*, ed. A. Rudin, Academic Press, San Diego, 1982, pp. 155-190.
2. J. T. Lai, D. Filla and R. Shea, *Macromolecules*, 2002, **35**, 6754-6756.
3. A. Shaira and D. Jaganyi, *J. Coord. Chem.*, 2014, **67**, 2843-2857.
4. C. R. Fenoli and C. N. Bowman, *Polym. Chem.*, 2014, **5**, 62-68.
5. L. W. Zhang, Y. H. Zhang and Y. M. Chen, *Eur. Polym. J.*, 2006, **42**, 2398-2406.
6. G. W. V. Cave and C. L. Raston, *J. Chem. Soc., Perkin Trans. 1*, 2001, **24**, 3258-3264.
7. A. Noor, S. C. Moratti and J. D. Crowley, *Chem. Sci.*, 2014, **5**, 4283-4290.
8. S. Schmatloch, A. M. J. van den Berg, A. S. Alexeev, H. Hofmeier and U. S. Schubert, *Macromolecules*, 2003, **36**, 9943-9949.
9. B. Morgan and O. Lahav, *Chemosphere*, 2007, **68**, 2080-2084.
10. R. W. Lewis, R. A. Evans, N. Malic, K. Saito and N. R. Cameron, *Polym. Chem.*, 2017, **8**, 3702-3711.
11. ASTM E2408-04, *Standard Test Method for Relative Extensional Viscosity of Agricultural Spray Tank Mixes*, ASTM International, West Conshohocken PA, 2015
